# Trabectedin modulates the senescence-associated secretory phenotype and promotes cell death in senescent tumor cells by targeting NF-κB

**DOI:** 10.18632/oncotarget.24961

**Published:** 2018-04-13

**Authors:** Simona Camorani, Laura Cerchia, Monica Fedele, Eugenio Erba, Maurizio D’Incalci, Elvira Crescenzi

**Affiliations:** ^1^ Istituto per l’Endocrinologia e l’Oncologia Sperimentale, Consiglio Nazionale delle Ricerche, 80131 Naples, Italy; ^2^ Dipartimento di Oncologia, IRCCS Istituto Di Ricerche Farmacologiche Mario Negri, 20156 Milan, Italy

**Keywords:** therapy-induced senescence, SASP, trabectedin, apoptosis, NF-kappaB

## Abstract

Therapy-induced senescence is a major cellular response to chemotherapy in solid tumors. Senescent tumor cells acquire a secretory phenotype, or SASP, and produce pro-inflammatory factors, whose expression is largely under NF-κB transcriptional control. Secreted factors play a positive role in driving antitumor immunity, but also exert negative influences on the microenvironment, and promote tumor growth and metastasis. Moreover, subsets of cancer cells can escape the senescence arrest, driving tumor recurrence after treatments. Hence, removal the senescent tumor cells, or reprogramming of the senescent secretome, have become attractive therapeutic options.

The marine drug trabectedin was shown to inhibit the production of pro-inflammatory mediators by tumor-infiltrating immune cells and by myxoid liposarcoma cells. Here, we demonstrate that trabectedin inhibits the SASP, thus limiting the pro-tumoral activities of senescent tumor cells *in vitro*. We show that trabectedin modulates NF-κB transcriptional activity in senescent tumor cells. This results in disruption of the balance between antiapoptotic and proapoptotic signals, and sensitization of cells to Fas-mediated apoptosis. Further, we found that trabectedin inhibits escape from therapy-induced senescence, at concentrations that do not affect the viability of bulk tumor population.

Overall, our data demonstrate that trabectedin has the potential to inhibit multiple detrimental effects of therapy-induced senescence.

## INTRODUCTION

Therapy-induced senescence (TIS or therapy-induced premature senescence) is a major cellular response to chemotherapy and radiotherapy in solid tumors [1, 2]. Premature senescent cells remain viable and metabolically active, and show distinct phenotypic features, such as morphological changes, senescence-associated beta-galactosidase activity, irreparable DNA damage and a permanent cell cycle arrest [2]. This anti-proliferative effect of TIS has provided the rationale for the development of pro-senescence therapies in cancer [3]. However, the acquisition of a senescent phenotype, in response to DNA-damaging chemotherapy, induces a gradual production of cytokines, chemokines, growth factors and matrix remodelling proteases, which affect the same senescent cells (cell-autonomous signaling) and the neighboring cells (cell non-autonomous signaling) [4]. Secretion of these factors has been termed senescence-associated secretory phenotype or SASP [5]. Experimental data obtained in the last years have revealed that NF-κB acts as a critical regulator of the SASP [6, 7]. SASP factors carry out several functions: they reinforce the senescence growth arrest [8], stimulate clearance of senescent cells by immune effectors [9], activate anti-tumor immunity [10], and modulate the cross-talk between senescent cells and the microenvironment. This cross-talk is responsible for several harmful effects of the SASP, such as promotion of tumor cell growth and invasion, stimulation of angiogenesis, development of resistance to chemotherapy [11]. In addition, although replicative senescence in normal cells imposes a permanent cell cycle exit, drug-induced senescent tumor cells spontaneously escape senescence and reenter the cell cycle [12, 13]. Hence, induction of TIS appears to have both favorable and detrimental effects, and new therapies should seek to reprogram the senescent secretome [14] or to eliminate senescent tumor cells [15].

Trabectedin (Yondelis or Ecteinascidin-743) is an anticancer agent isolated from the tunicate *Ecteinascidia turbinata*, presently produced by a semi-synthetic process. Being effective against several preclinical tumor models [16] trabectedin was investigated in the clinic and it is currently used for the treatment of patients with soft tissue sarcomas after failure of doxorubicin and ifosfamide [17] and of relapsing platinum-sensitive ovarian cancer patients in combination with pegylated liposomal doxorubicin [18, 19], and ongoing studies suggest it is effective also against other solid malignancies, including breast cancer [20]. Previous studies have revealed a complex mechanism of action for trabectedin. The drug binds to the minor groove of DNA, thus inducing a smooth bending of the helix toward the major groove [21]. Furthermore, trabectedin causes a gene- and promoter-dependent modulation of transcription [22]. Notably, at barely cytotoxic concentrations, trabectedin inhibits the production of proinflammatory mediators by innate immune cells and modulates the tumor microenvironment [23, 24]. The effects of trabectedin on non-cycling, premature senescent tumor cells and the SASP have not been investigated.

NF-κB is a family of transcription factors playing critical roles in immune-cell function, inflammation, apoptosis, cell cycle progression and DNA damage response [25]. In particular, the RelA/p65 subunit of NF-κB is a well-established activator of antiapoptotic genes and is regarded as a critical mediator of tumor cell survival and chemoresistance [26]. However, depending on the stimulus and cellular settings, RelA/p65 can repress antiapoptotic genes, such as Bcl-XL, actually enhancing, rather than inhibiting, apoptosis [27].

In this study we investigated the effects of trabectedin on drug-induced senescent tumor cells. We show that trabectedin modulates RelA/p65 transcriptional activity in senescent tumor cells. This results in repression of antiapoptotic genes and sensitization of cells to Fas-mediated apoptosis at high concentrations, and inhibition of the SASP at low concentrations. Accordingly, trabectedin counteracts the pro-tumoral activities of senescent tumor cells *in vitro*. These findings suggest that trabectedin can be used to manage the detrimental effects of TIS, and strongly support further *in vivo* investigation.

## RESULTS

### Effects of trabectedin on senescent tumor cells viability and apoptosis

In order to investigate the effects of trabectedin on premature senescent tumor cells, we induced senescence in the breast cancer cell line MCF-7 and in the lung cancer cell line A549. Both MCF-7 and A549 cells have wild-type p53, which is a pivotal mediator of cellular senescence [15]. Accordingly, both cell lines readily undergo senescence upon treatment with sublethal concentrations of doxorubicin, and have been previously characterized as a model of TIS in our lab [28, 6]. However, since trabectedin has been shown to induce cell death in a p53-independent manner [16, 29], we also analyzed MDA-MB-231 breast cancer cells, expressing mutant p53 (R280K). As illustrated in Supplementary Figure 1, senescent MCF-7, A549 and MDA-MB-231 cells showed morphologic alterations and positive staining for SA-beta-gal (Supplementary Figure 1A), persistent γ-H2AX foci (Supplementary Figures 1B and 2), accumulation of hypophosphorylated pRb and upregulation of p21CIP1 (Supplementary Figure 1C and 1D), cell cycle arrest (Supplementary Figure 1E). Furthermore, in order to confirm lack of cell division in senescent MDA-MB-231 cell line, we stained proliferating and senescent MDA-MB-231 cells with membrane dye PKH2. As shown in Supplementary Figure 1F, while proliferating cells showed a progressive decrease in PKH2 staining intensity, reflecting cell division, senescent cells fail to proliferate, and exhibited unchanged PKH2 staining intensity over a period of 8 days after release from cisplatin. In addition, no significant apoptosis was detected in senescent MDA-MB-231 cells (Supplementary Figure 1G). Finally, senescent MDA-MB-231 cells showed induction of cytokines characterizing the SASP (Supplementary Figure 1H). These data confirm the observation that TIS can be induced in cancer cells lacking functional p53 [2].

**Figure 1:**
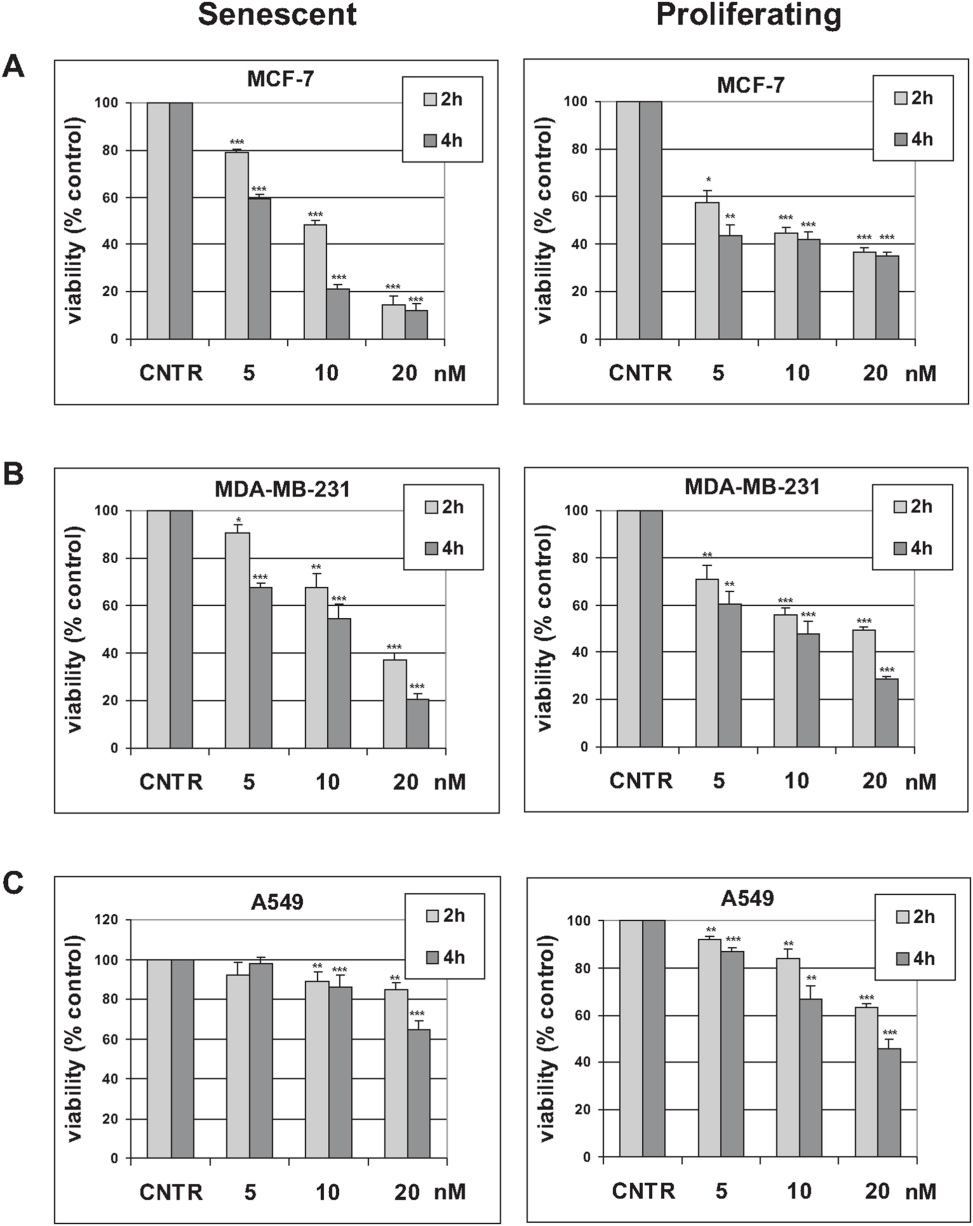
Effect of trabectedin on tumor cells viability Tumor cells were induced to undergo senescence by treatment with doxorubicin. Height **(A)**, five **(B)** or six **(C)** days after release from doxorubicin, senescent cells were treated with 5 to 20 nM trabectedin for indicated times. Proliferating tumor cells were also treated with 5 to 20 nM trabectedin for indicated times. Cell viability was determined 72h after trabectedin washout. Data are mean ± S.D. of one representative experiment out of two (MDA-MB-231) or three (MCF-7 and A549) independent experiments, performed in triplicate.

Proliferating and senescent cells were treated with trabectedin, using a range of concentrations and incubation times previously used to induce apoptosis in cancer cells [29], and cell viability was assessed 72 hours later. As shown in Figure 1, trabectedin induced loss of viability in both senescent and proliferating cells that showed similar susceptibility to the drug, whereas significant differences in sensitivity were observed between different cell lines, with both breast cancer cell lines being more sensitive than A549 cells (Figure 1). Actual loss of senescent cells after trabectedin exposure was confirmed by cell counting (Supplementary Figure 3). The same effect of trabectedin on senescent cells viability was observed in MCF-7 cells induced to undergo premature senescence by hydrogen peroxide (Supplementary Figure 4).

Trabectedin has been shown to slow the rate of progression through S phase in proliferating tumor cell lines and to induce an accumulation in late S and G2/M [20]. Hence, we assessed the effects of trabectedin on cell cycle. 24h after trabectedin treatment, a significantly inhibition of 5-bromo-2-deoxyuridine (BrdU) incorporation (Supplementary Figure 5A; quantified in Supplementary Figure 5B) and a G2/M increase (Supplementary Figure 5C) was observed in all proliferating cells. Interestingly, de novo BrdU incorporation was induced in senescent MDA-MB-231 cells, suggesting that the drug might stimulate abortive cell cycle re-entry in the absence of p53-dependent checkpoints (Supplementary Figure 5A and 5B).

It has been previously demonstrated that trabectedin sensitizes cancer cells to Fas-mediated cell death [29]. In addition, we previously showed that induction of premature senescence renders cancer cells prone to Fas-mediated apoptosis [6]. Hence, in order to confirm that the observed loss of viability is related to activation of the Fas pathway, we analyzed the expression of Fas on senescent MCF-7 cells. In line with previous observations [6], senescent MCF-7 cells expressed Fas on their surface, as assessed by flow cytometric analyses, and expression was significantly increased by trabectedin treatment (Supplementary Figure 6A). We next analyzed Caspase-8 activation. As shown in Supplementary Figure 6B, trabectedin induced activation of Casp-8 in all senescent cells tested, as indicated by progressive disappearance of intermediate p43/p41 fragments. Cleavage of Casp-8 to the fully processed p18 form was further confirmed in MCF-7 cells by flow cytometry (Supplementary Figure 6C), and in MDA-MB-231 cells by western blot (Supplementary Figure 6D). Finally, induction of mitochondrial membrane depolarization was analyzed by staining with TMRE in MCF-7 cells (Supplementary Figure 6E). Trabectedin-dependent cleavage of Casp-8 was also detected in hydrogen peroxide-induced senescent MCF-7 cells (Supplementary Figure 6F). Hence, trabectedin activates an apoptotic pathway in premature senescent tumor cells. Upregulation of Fas expression will be further analyzed below.

### Trabectedin counteracts multiple negative effects of TIS

**Figure 2:**
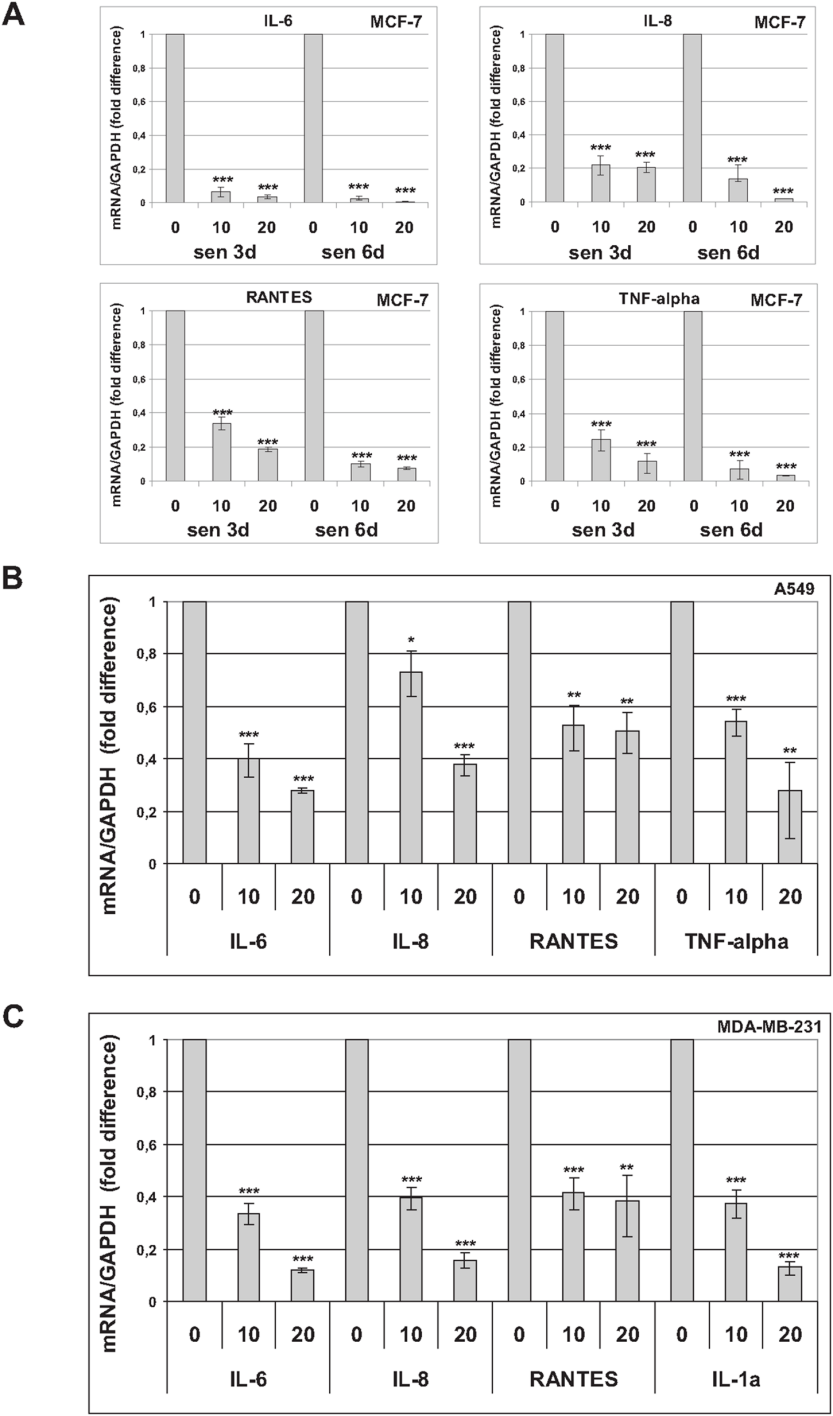
Trabectedin reduces the SASP Cytokines and chemokines were analyzed by real-time PCR in premature senescent tumor cells, 72h after drug washout. **(A)** Three and six days after release from doxorubicin, senescent MCF-7 cells were treated with 10 or 20 nM trabectedin for 1h. **(B)** Seven days after release from doxorubicin, senescent A549 cells were treated with 10 or 20 nM trabectedin for 2h. **(C)** Seven days after release from cisplatin, senescent MDA-MB-231 cells were treated with 10 or 20 nM trabectedin for 1h. Data are mean ± S.D. of one representative experiment out of two to four independent experiments performed in triplicate.

**Figure 3:**
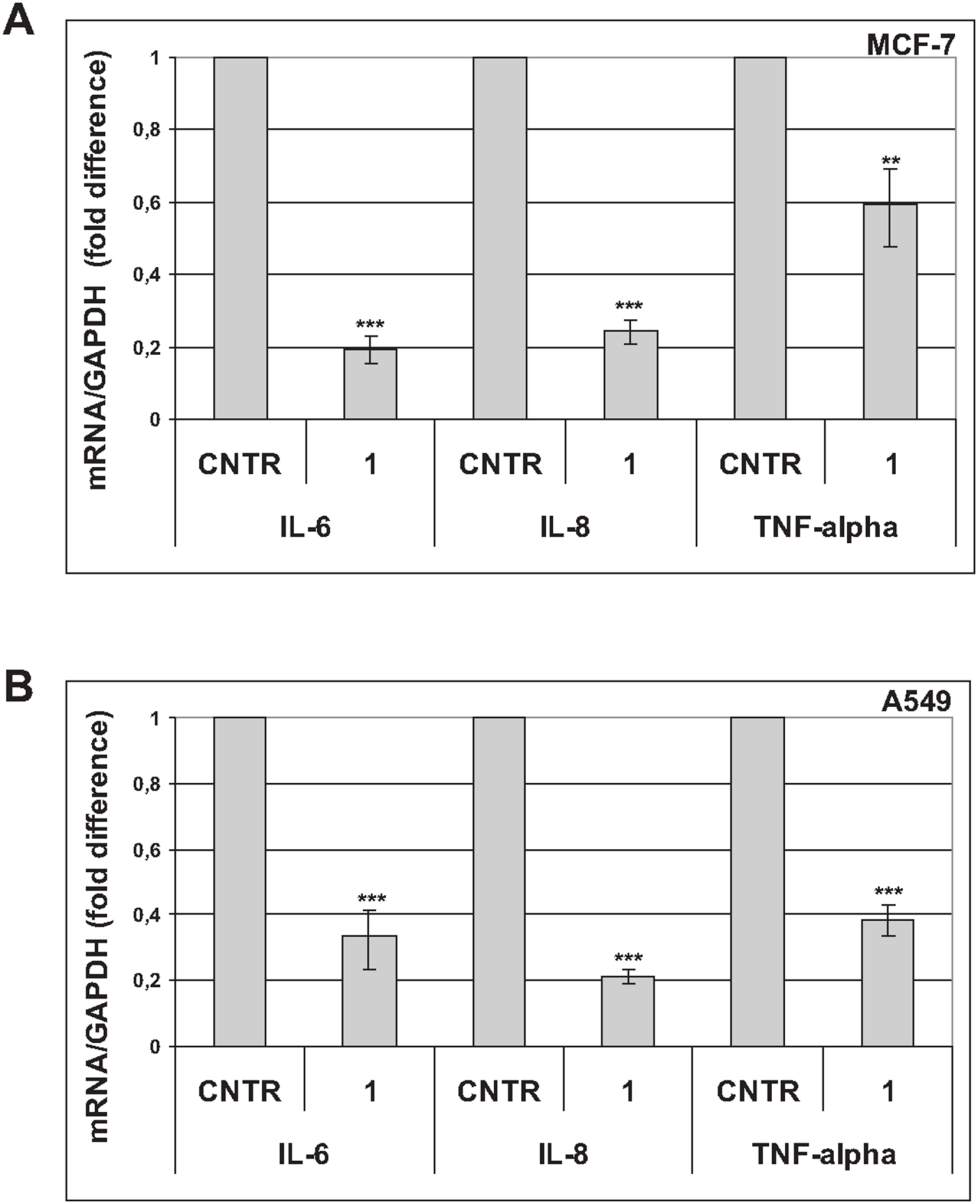
Trabectedin reduces the SASP Seven days after release from doxorubicin, senescent MCF-7 **(A)** and senescent A549 **(B)** cells were treated with 1 nM trabectedin for 24h. Expression of cytokines was analyzed by real-time PCR 6 days after drug washout. Data are mean ± S.D. of one representative experiment out of two independent experiments performed in triplicate.

It has been demonstrated that trabectedin inhibits the production of proinflammatory mediators by innate immune cells [23, 24]. Since senescent cells acquire a largely proinflammatory secretion profile, we investigated the effects of trabectedin on a subset of cytokines and chemokines whose expression is significantly increased in premature senescent tumor cells (Supplementary Figures 1H and 7). As SASP develops gradually, the expression of proinflammatory cytokines (IL-6, IL-8, TNF-alpha) and chemokines (RANTES, CXCL10) was first analyzed by real-time PCR 3 days and 6 days after induction of senescence in MCF-7 cells. As shown in Figure 2A, trabectedin significantly reduced the SASP at all concentrations tested. In addition, the effect of the drug was not affected by the degree of senescence, since it was similarly observed at different times during the development of a fully senescent phenotype. The ability of trabectedin to reduce the SASP was then confirmed in premature senescent A549 (Figure 2B) and MDA-MB-231 cells (Figure 2C). Most importantly, significant downregulation of SASP factors was also observed after exposure to a non-cytotoxic concentration of trabectedin (1 nM) for 24h, and such inhibition was maintained for several days after trabectedin washout (Figure 3). These data collectively demonstrate that trabectedin negatively modulates the SASP in premature senescent tumor cells. Interestingly, among analyzed chemokines, CXCL10 and CXCL12 were generally less affected by treatments (Supplementary Figure 8), suggesting that different SASP components exhibit different sensitivity to the drug.

**Figure 4:**
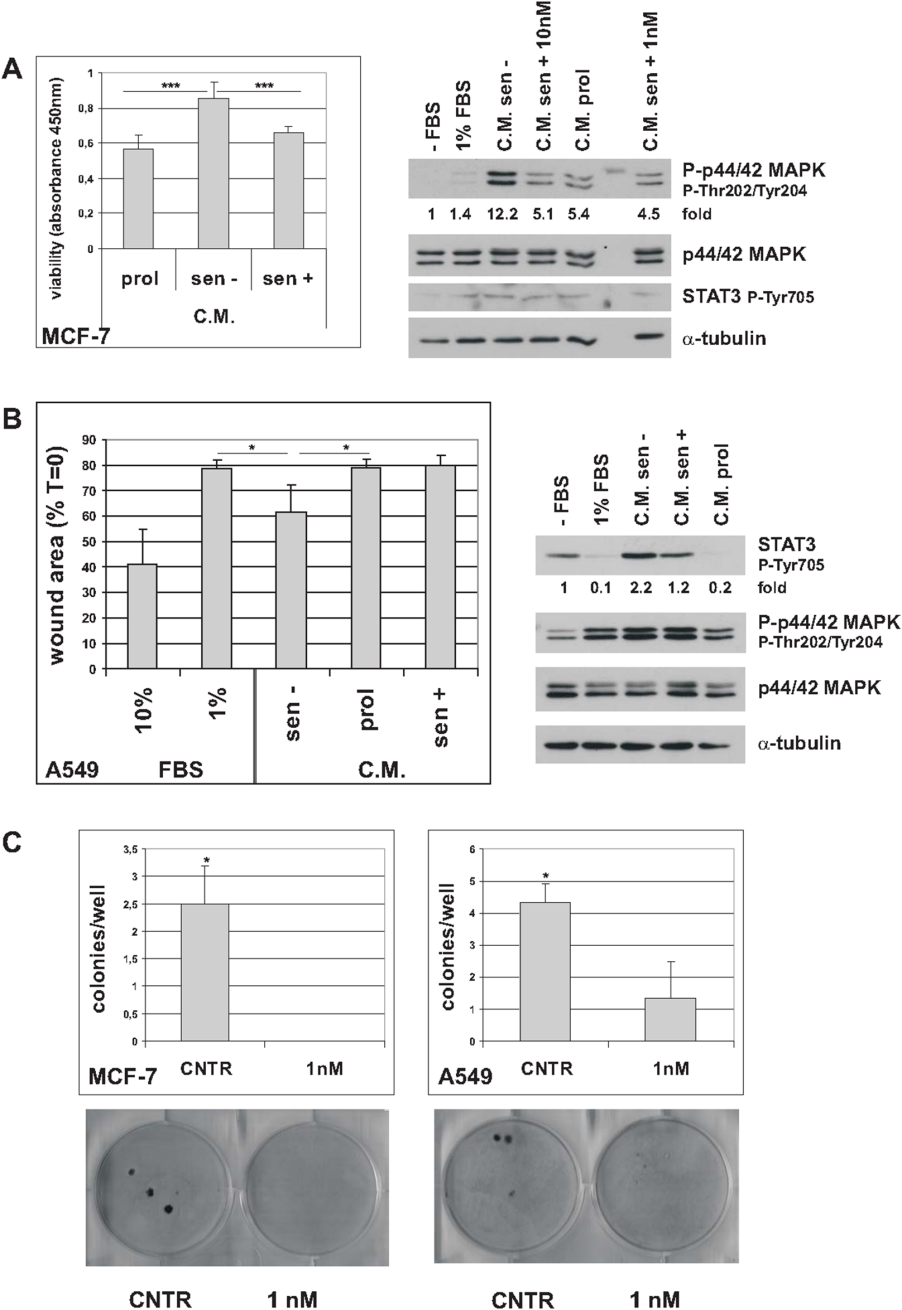
Trabectedin hinders harmful effects of senescence **(A)** *Left*: Proliferating MCF-7 cells were incubated with conditioned media (C.M.) from untreated senescent cells (sen-), 10 nM treated senescent cells (sen+) or proliferating cells (prol) and cell viability was determined after 72h. Data are mean ± S.D. of one representative experiment out of three independent experiments performed in triplicate. *Right*: Proliferating MCF-7 cells were serum-starved for 24h and then stimulated with medium supplemented with 1% FBS or C.M. from untreated senescent cells (C.M. sen -), 10 nM treated senescent cells (C.M. sen + 10 nM), proliferating cells (C.M. prol) or 1 nM treated senescent cells (C.M. sen + 1 nM). Expression of phospho-p44/42MAPK proteins and phospho-STAT3 was analyzed after 1h stimulation. Filters were stripped and reprobed with anti p44/42MAPK and anti-α-tubulin antibodies as a loading control. Phospho-p44/42MAPK levels, normalized to total p44/42MAPK, are reported as fold change of serum-starved sample. **(B)** *Left*: effects of C.M. from untreated senescent A549 cells (sen -) or treated senescent A549 cells (sen +) on A549 migration, analyzed by wound healing assay. C.M. from proliferating cells (prol) was used as negative control. The area of a wound was analyzed with the MRI Wound Healing Tool (ImageJ software). Data are mean ± S.D. of one representative experiment out of four independent experiments performed in duplicate. *Right*: Proliferating A549 cells were serum-starved for 24h and then stimulated with medium supplemented with 1% FBS or C.M. from untreated 7 days senescent cells (C.M. sen -), 10 nM treated 7 days senescent cells (C.M. sen +) or proliferating cells (C.M. prol). Expression of phospho-STAT3 and phospho-p44/42MAPK proteins was analyzed after 1h stimulation. Filters were stripped and reprobed with anti p44/42MAPK and anti-α-tubulin antibodies as a loading control. Phospho-STAT3 levels, normalized to the relative α-tubulin levels, are reported as fold change of serum-starved sample. **(C)** Effect of trabectedin on senescence escape. Four days after release from doxorubicin, senescent MCF-7 and A549 cells were treated with 1 nM trabectedin for 24h. Colonies that evaded the senescent growth arrest were stained and counted. Upper panels: quantification of the colony escape assay. Data are mean ± S.D. of one representative experiment out of three independent experiments performed in triplicate. Lower panels: representative image of the colony escape assay.

The SASP mediates many harmful effects of senescence [4, 11]. Since trabectedin appears to reduce and modulate the senescent secretome, we investigated the effects of conditioned media (C.M.) from trabectedin-treated or untreated senescent tumor cells on MCF-7 cells viability. As shown in Figure 4A *left*, C.M. from senescent cells significantly increased the viability of MCF-7 cells, as compared to C.M. from control, proliferating cells. This stimulatory effect was nullified using the C.M. obtained from senescent cells 6 days after trabectedin treatment. In addition, western blot analyses of proliferating MCF-7 cells, serum-starved and then stimulated with C.M., showed that phosphorylation of p44/42MAPK was strongly induced by C.M. from senescent cells, and substantially reduced using the C.M. from trabectedin-treated senescent cells (Figure 4A, *right*). In line with data shown in Figure 2 and Figure 3, p44/42MAPK phosphorylation was equally reduced by exposing senescent cells to either high concentration trabectedin for a short time (10 nM, 1h) or low concentration trabectedin for longer time (1 nM trabectedin, 24h). STAT3 phosphorylation was not affected by any treatment in MCF-7 cells (Figure 4A, right). Furthermore, in wound healing assays, A549 migration was induced by C.M. from senescent cells, as compared to both C.M. from proliferating cells and control medium supplemented with 1% FBS (Figure 4B and Supplementary Figure 8). In contrast, C.M. from trabectedin-treated senescent cells did not sustain cell migration (Figure 4B and Supplementary Figure 9). It is worth noting that proliferation rate of A549 cells was not influenced by C.M., either from untreated or from trabectedin-treated senescent cells (Supplementary Figure 9C), indicating that the observed effects on cell migration were not a consequence of a general inhibition of cell proliferation. Accordingly, also C.M. from cells silenced for RelA/p65, which show a low SASP phenotype [6], did not affect the proliferation of A549 cells (Supplementary Figure 9C). In line with these findings, western blot analysis of proliferating A549 cells, serum-starved and then stimulated with C.M. from senescent cells, showed no change in p44/42MAPK phosphorylation (Figure 4B, *right*). On the other hand, C.M. from senescent A549 cells induced a robust STAT3 phosphorylation, which was reduced using the C.M. from trabectedin-treated senescent cells (Figure 4B, *right*). These results are consistent with the scratch wound healing data and suggest that STAT3 plays a relevant role in paracrine SASP signaling in A549 cells. Since STAT3 is a well known downstream effector of IL-6 and TNF-alpha, these data further support the ability of trabectedin to inhibit proinflammatory cytokines secretion from senescent tumor cells. On the whole, these data demonstrate that trabectedin reduces the SASP, and reduces its biological effects *in vitro*. The observed differences between MCF-7 and A549 cells in response to C.M., i.e. stimulation of proliferation and p44/42MAPK phosphorylation vs. stimulation of migration and STAT3 phosphorylation, are consistent with variations in SASP composition between cell types, leading to diverse downstream effects [4].

One positive function of the SASP is to orchestrate immune surveillance. In order to investigate a potential effect of trabectedin on recruitment of innate immune cells, we used C.M. from proliferating, trabectedin-treated or untreated senescent tumor MCF-7 cells to drive THP-1 monocytic cell line migration in a transwell assay. As shown in Supplementary Figure 10, C.M. from senescent cells attracted THP-1 cells into the bottom chamber, as compared to C.M. from proliferating cells. This stimulatory effect was enhanced using the C.M. obtained from senescent cells previously exposed to trabectedin. This observation suggests that trabectedin might positively modulate the recruitment of innate immune cells.

Cancer stem cells can escape TIS [12, 13]. Hence, we investigated the effects of trabectedin on senescence escape. Premature senescent MCF-7 and A549 cells were treated with 1 nM trabectedin for 24h and extensively washed. After 15 to 30 days, colonies that evaded the senescent growth arrest were counted. As shown in Figure 4C, trabectedin treatment completely inhibited escape from senescence in MCF-7 cells, and significantly reduced evasion in A549 cells. In addition, A549 colonies formed in trabectedin-treated plates were sensibly smaller than those escaped in controls.

### Trabectedin modulates NF-κB transcriptional activity in premature senescent tumor cells

**Figure 5:**
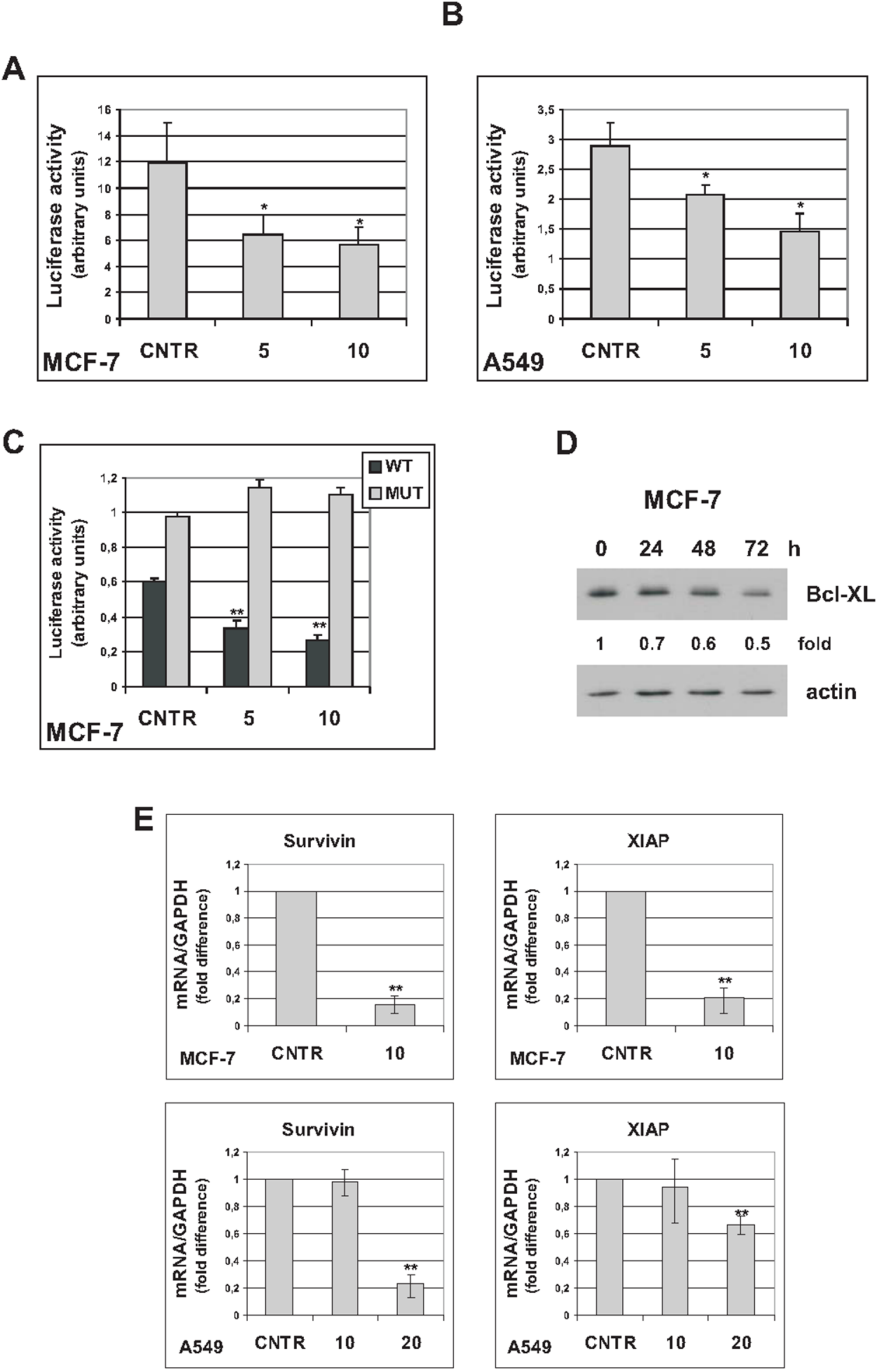
Trabectedin represses NF-κB reporter plasmid activity **(A)** Five days after release from doxorubicin, senescent MCF-7 were transiently cotransfected with Ig-κB luciferase reporter and Renilla luciferase reporter plasmids. After 24h, cells were either untreated (CNTR) or treated with trabectedin (5 and 10 nM) for 1h. Luciferase activity was measured 24h after drug washout. Data are mean ± S.D. of one representative experiment out of two independent experiments performed in triplicate. **(B)** Seven days after release from doxorubicin, senescent 549 cells were cotransfected as in (A), and treated with trabectedin 5 and 10 nM for 2h. **(C)** Four days after release from doxorubicin, senescent MCF-7 cells were transiently cotransfected with either Bcl-XL κB-luciferase reporter plasmid (WT) or Bcl-XL-Δ-κB-luciferase reporter plasmid, with mutated κB sites (MUT), and Renilla luciferase reporter plasmid. After 24h, cells were treated as in (A). **(D)** Four days after release from doxorubicin, senescent MCF-7 cells were treated with 5 nM trabectedin for 1h. Bcl-XL protein was detected for up to 72h after drug washout. Filters were stripped and re-probed with anti-actin antibody. Bcl-XL levels, normalized to the relative actin levels, are reported as fold change of untreated cells. **(E)** Senescent MCF-7 cells (seven days after release from doxorubicin) were treated with 10 nM trabectedin for 1h; senescent A549 cells (six days after release from doxorubicin) were treated with 10 or 20 nM trabectedin for 2h. Expression of Survivin and XIAP was estimated by real-time PCR 72h after drug washout. Data are mean ± S.D. of one representative experiment out of two independent experiments performed in triplicate.

The transcription factor NF-κB is activated in senescent tumor cells and plays a critical role in controlling the SASP [6, 7]. In addition, NF-κB plays a protective role in cancer cell survival [26]. Since trabectedin reduces cell viability and inhibits the SASP, we investigated whether NF-κB activity is modulated in senescent cells exposed to trabectedin, by luciferase reporter assay. As shown in Figure 5A and 5B, trabectedin treatment resulted in inhibition of an Ig-κB luciferase reporter plasmid, in both MCF-7 and A549 senescent cells, while having the opposite effect in proliferating cells (Supplementary Figure 11). It has been demonstrated that, depending on the apoptotic stimulus and the cellular context, RelA/p65 can actively inhibit transcription, and under such conditions represses antiapoptotic genes, such as Bcl-XL [27]. To address whether such a mechanism also operates in senescent tumor cells exposed to trabectedin, we transfected senescent MCF-7 with a luciferase reporter, controlled by the human Bcl-XL promoter, with either a functional κB-site or with mutated κB-site [27]. As shown in Figure 5C, wild-type (WT), not mutant (MUT), Bcl-XL-κB luciferase reporter activity was inhibited in trabectedin-treated cells. These results were also confirmed at protein level, by western blot analysis of MCF-7 treated with 5 nM trabectedin and analyzed for up to 72h that shows progressive decrease in Bcl-XL (Figure 5D). Trabectedin reduced Bcl-XL protein levels also in hydrogen peroxide-induced senescent MCF-7 cells (Supplementary Figure 6F). In line with these observations, also Survivin and XIAP, endogenous NF-κB antiapoptotic target genes, were downregulated in senescent MCF-7 and A549 cells exposed to cytotoxic concentrations of trabectedin (Figure 5E). It is unlikely that the observed effects are a consequence of any non-specific inhibition of gene expression due to cytotoxicity, since no reduction in Bcl-XL-MUT-κB luciferase expression or Renilla luciferase expression was observed in trabectedin-treated cells. Furthermore, a RelA/p65-dependent upregulation of pro-apoptotic Fas/Cd95 gene was observed in the same experimental conditions (see below). These data strongly support the importance of NF-κB in mediating cytotoxicity of the drug in premature senescent tumor cells.

### RelA/p65 mediates trabectedin-dependent apoptosis in premature senescent tumor cells

**Figure 6:**
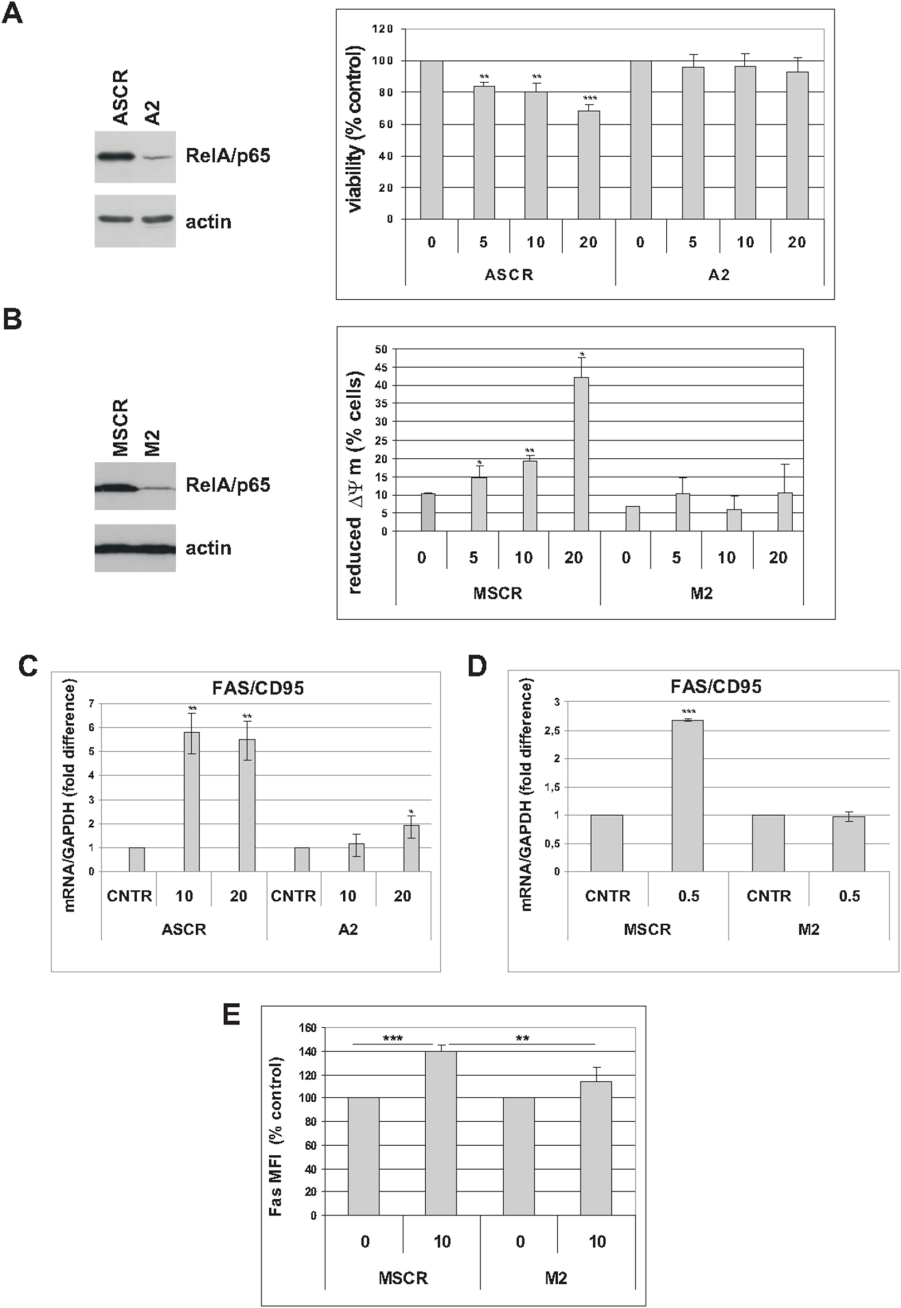
RelA/p65 mediates cytotoxic effects of trabectedin in senescent tumor cells **(A)** *Left*: five days after release from doxorubicin, expression of RelA/p65 was analyzed in senescent A549 cells, scrambled (ASCR) or RelA-interfered (A2). Filters were stripped and reprobed with anti-actin antibody as a loading control. *Right*: five days after release from doxorubicin, senescent ASCR or A2 cells were treated with 0 to 20 nM trabectedin for 4h. Cell viability was determined 72h after drug washout. Data are mean ± S.D. of one representative experiment out of two independent experiments performed in triplicate. **(B)** Left: four days after release from doxorubicin, expression of RelA/p65 was analyzed in senescent MCF-7 cells, scrambled (MSCR) or RelA-interfered (M2). Filters were stripped and reprobed with anti-actin antibody as a loading control. Right: four days after release from doxorubicin, senescent MSCR or M2 cells were treated with 0 to 20 nM trabectedin for 1h. Apoptosis was estimated by TMRE staining 72h after drug washout. Data are mean ± S.D. of one representative experiment out of three independent experiments. **(C)** Five days after release from doxorubicin, senescent ASCR and A2 cells were treated with 0 to 20 nM trabectedin for 2h. Fas expression was estimated by real-time PCR 48h after drug washout. Data are mean ± S.D. of one representative experiment out of two independent experiments performed in triplicate. **(D)** Four days after release from doxorubicin, senescent MSCR and M2 cells were treated with 0.5 nM trabectedin for 24h and Fas expression was estimated by real-time PCR. Data are mean ± S.D. of one representative experiment out of two independent experiments performed in triplicate. **(E)** Senescent MSCR and M2 cells were treated with 10 nM trabectedin for 1h and analyzed for Fas expression 72h after drug washout. Levels of surface Fas are expressed as relative mean fluorescence intensity (MFI) minus background fluorescence of isotype-matched control. Data are mean ± S.D. of one representative experiment out of two independent experiments performed in triplicate.

To further substantiate the role of NF-κB in mediating the cytotoxic effects of trabectedin in premature senescent tumor cells, we analyzed A549 and MCF-7 cells in which expression of RelA/p65 was knocked-down by small hairpin RNA (A2 and M2, respectively, Figure 6), as compared with scramble infected controls (ASCR and MSCR, respectively, Figure 6) [6]. As shown in Figure 6A, silencing of RelA/p65 renders A549 senescent cells resistant to trabectedin. Staining with TMRE, showing decreased mitochondrial membrane depolarization in interfered senescent MCF-7 cells, confirmed impaired trabectedin-dependent apoptosis in the absence of RelA/p65 (Figure 6B).

NF-κB directly regulates Fas transcription [30]. Since trabectedin modulates NF-κB transcriptional activity, we wondered whether upregulation of Fas in senescent tumor cells (Supplementary Figure 6 and ref. 29) could also depend on NF-κB. As shown in Figure 6C and 6D, Fas expression was significantly increased in response to trabectedin in ASCR and MSCR control cells, and less upregulated in RelA/p65-interfered cells. These results were further substantiated by flow cytometric staining of surface Fas protein (Figure 6E). These data, as a whole, indicate that NF-κB mediates trabectedin cytotoxicity in premature senescent tumor cells.

## DISCUSSION

Therapy induced senescence is a major cellular response to genotoxic chemotherapy and radiation therapy [2]. Senescent tumor cells develop a secretory phenotype that elicits various positive effects such as prolonged cell cycle arrest, immune clearance, and activation of anti-tumor immunity [4]. In addition, induction of TIS by low dose chemotherapy is potentially less toxic and may reduce side effects in patients. Still, senescent cells also exert protumorigenic activities, similarly mediated by the SASP. Hence, therapeutic strategies to clear senescent cells or to reprogram the SASP have become of great interest [14, 15]. In this manuscript we show that the marine drug trabectedin activates an apoptotic pathway in drug-induced senescent tumor cells at high concentrations, and suppresses the SASP and the escape at low, non-cytotoxic concentrations, thus counteracting multiple negative effects of premature senescence. The ability of trabectedin to modulate the production of inflammatory mediators has been previously described in macrophages, where it has been related to suppression of differentiation and viability [31], and also in specific tumor types, such as myxoid liposarcoma [32], where the capacity to modulate the microenvironment contributed to the activity of the drug. Here we show that in premature senescent tumor cells trabectedin causes a marked decrease in the expression of proinflammatory cytokines (IL-6, IL-8, TNF-alpha), which play critical roles in tumor growth and metastasis [33]. Accordingly, conditioned media obtained from trabectedin-treated senescent tumor cells loose the capacity to sustain either viability or migration in the corresponding proliferating cells. Importantly, SASP inhibition is independent from cytotoxicity, being already achieved at a very low concentration (1 nM), and is maintained for several days after the cessation of treatment.

One positive function of the SASP is to orchestrate the crosstalk between senescent cells and immune cells, thus regulating the recruitment of immune effectors to the tumor site and the activation of immune surveillance [34, 35]. Our experiments show that trabectedin differentially modulates diverse SASP factors. For instance, real-time PCR analyses revealed that expression of the chemokine CXCL10 (IP-10) was largely unaffected by trabectedin. CXCL10 is a potent monocyte and T-cell chemoattractant, promotes generation of effector T cells, and has been shown to exert tumor-suppressive functions in several cancer types [36]. Interestingly, conditioned media from trabectedin-treated senescent MCF-7 cells show an enhanced ability to stimulate THP-1 monocytic cell migration in transwell assays. These observations indicate that trabectedin does not simply inhibit, but rather modulates the SASP, and suggest a potential role for trabectedin in tuning anti-tumor immunity in the senescent context, which requires *in vivo* studies to be properly addressed. Another beneficial function of the SASP is paracrine stimulation of senescence in neighboring cells [37]. However, preliminary evidence from our laboratory suggests that paracrine senescence is not induced in our cell system.

NF-κB plays a critical role in regulating the SASP, and most of the analyzed cytokines and chemokines are secreted in a NF-κB-dependent manner [7]. As discussed below, trabectedin modulates NF-κB activity in premature senescent tumor cells. However, it is important to note that at least one relevant cytokine, IL-6, which in our cell system is not under NF-κB transcriptional control [6] is also significantly downregulated by trabectedin. This finding suggests that the drug modulates the SASP by inhibiting multiple transcription factors, which is in line with the ability of trabectedin to act as a broad spectrum inhibitor of active transcription [38]. It has been shown that depletion of C/EBPβ substantially reduces IL-6 secretion during cellular senescence [11]. Interestingly, trabectedin has been shown to both upregulate C/EBPβ protein and to modulate its binding activity to target promoters in myxoid liposarcoma cells [32]. We did not detect alterations in C/EBPβ protein levels after treatment with trabectedin (Supplementary Figure 12); however, a detailed investigation of C/EBPβ ability to bind and to transactivate proinflammatory genes will be required in order to fully dissect its role as mediator of trabectedin in premature senescent tumor cells.

In a previous screening of small molecule drugs, trabectedin was identified as a potent inhibitor of canonical NF-κB signaling pathway [39]. Here, we demonstrate that trabectedin not only decreases NF-κB transcriptional activity (which is likely responsible for reduced transcription from endogenous cytokines and chemokines promoters), but also induces a RelA/p65-dependent active repression of antiapoptotic genes. In particular, we show that trabectedin represses a Bcl-XL-luciferase reporter plasmid in a κB-dependent manner, and downregulates Bcl-XL protein. In agreement with this proapoptotic function of NF-κB, we found that interference with RelA/p65 prevented trabectedin-dependent apoptosis in premature senescent tumor cells. Interestingly, it has been recently demonstrated that, among Bcl-2 family members, Bcl-XL and Bcl-W are essential for viability of senescent cells [40].

It has been shown that atypical stimuli, such as daunorubicin, can convert RelA/p65 from antiapoptotic to proapoptotic mediator [27] possibly as a result of torsional stress generated by DNA intercalation [41]. In this regard, it may be noted that trabectedin induces changes in DNA backbone torsional angles [42]. However, the exact signal(s) generated from damaged DNA, responsible for the activation of “repressor RelA/p65”, is still not clear.

Trabectedin was shown to precondition cancer cells to Fas-mediated death [29]. We confirm that trabectedin activates an extrinsic apoptotic pathway in senescent tumor cells, as demonstrated by enhanced surface Fas, activation of caspase-8 and downstream mitochondrial depolarization. Activation of Fas signaling involves the formation of large Fas aggregates. Since both Fas overexpression by itself, as well as different chemotherapeutic agents, can elicit Fas clustering in the absence of its ligand [43], it is highly likely that trabectedin triggers a FasL-independent activation of the death receptor in premature senescent tumor cells. Importantly, we demonstrate that NF-κB is responsible for overexpression of Fas upon trabectedin treatment. Indeed, interference with RelA/p65 significantly reduced trabectedin-dependent Fas upregulation, and cell death. On the whole, these data suggest that, in senescent tumor cells exposed to trabectedin, the balance of proapoptotic and antiapoptotic signals is modulated by NF-κB complexes, which repress antiapoptotic genes, such as Bcl-XL, and induce proapoptotic genes, such as Fas.

Finally, we demonstrate that trabectedin inhibits escape from senescence in tumor cells, at concentrations that do not affect viability of bulk population. Previous studies have revealed that a small subset of tumor cells is able to evade senescence induced by different anticancer drugs [12, 13]. Cells that escape TIS display an aggressive phenotype, characterized by cancer stem cell-like (CSC) and epithelial-mesenchymal transition (EMT) features [44, 45], and have been proposed to drive tumor recurrence after therapies [46]. Although the mechanisms responsible for senescence escape, as well as the molecular pathways allowing tumor cells to re-enter the cell cycle, are not fully understood, the activation of antiapoptotic signals, required for long-term CSC survival after treatments, has been postulated by several investigators. For instance, Survivin inhibits apoptosis and promotes senescence escape in H1299 lung cancer cells following chemotherapy [47]. Tumor cells that escape senescence also rely on Bcl-XL and Mcl-1 signaling [48]. Interestingly, Survivin, Bcl-XL and Mcl-1 are well-known NF-κB target genes [27, 49, 50]. Here we have shown that RelA/p65 exerts a trabectedin-dependent repressive function on Bcl-XL promoter in premature senescent tumor cells. We also found that RelA/p65-interfered cells show increased basal frequency of evasion from senescence, when compared to scramble shRNA cells (Crescenzi E, unpublished data). Based on these findings, it is plausible to speculate that NF-κB represses Bcl-XL gene expression, and possibly other antiapoptotic genes, both in basal conditions and in response to trabectedin, thus modulating CSC survival in TIS.

In conclusion, we demonstrated that trabectedin modulates the secretome and promotes cell death in senescent tumor cells, and identified NF-κB as a critical mediator of the drug's effects, suggesting the possibility to pharmacologically modulate the SASP and NF-κB in premature senescent tumor cells. We are currently developing a syngeneic mouse model of drug-induced senescence to study the *in vivo* effects of trabectedin on TIS to confirm such hypothesis in the next future. Moreover, our data suggest that, by targeting CSC, trabectedin could prevent the re-growth of neoplastic cell populations after chemotherapy, and may be effective in the long-term. Studies are underway in our lab to investigate the effects of trabectedin on CSC and to clarify the role of NF-κB in senescence escape.

## MATERIALS AND METHODS

### Cell culture, biological reagents and trabectedin treatments

A549, MCF-7 and MDA-MB-231 cells were obtained from American Type Culture Collection and cultured according to its instructions. All media were supplemented with 10% fetal bovine serum (FBS). The cell culture media and reagents were purchased from Sigma-Aldrich (Sigma-Aldrich, Milan, Italy). Doxorubicin hydrochloride (Sigma-Aldrich, Milan, Italy) was dissolved in sterile water. Cis-diammineplatinum (II) dichloride (Sigma-Aldrich, Milan, Italy) was dissolved in PBS. Tetramethylrhodamine-ethyl-ester (TMRE) (Thermo Fisher Scientific, MA, USA) was dissolved in dimethylsulfoxide.

Trabectedin was supplied by PharmaMar (Colmenar Viejo, Madrid, Spain), dissolved in dimethylsulfoxide to 1mM, and stored at -20°C. Cells were treated with trabectedin as indicated in Figure Legends, extensively washed, and incubated under standard conditions until analyzed.

### Induction of premature senescence and senescence associated-beta-galactosidase (SA-β-gal) activity

Unless otherwise stated, senescence was induced by treating cells with the DNA-damaging agent doxorubicin (200 nM for MCF-7 and MDA-MB-231 cells, and 600 nM for A549 cells) for 72h, as previously described [28]. Where indicated, Cisplatin (10μM for 24h) was used to induce senescence in MCF-7 and MDA-MB-231 cells. To allow the development of a fully senescent phenotype, cells were analyzed from 3 to 30 days after replating. Staining for SA-β-gal was performed as previously described [51].

### Cell viability

Cell viability was determined by water-soluble tetrazolium salt method, using either MTS assay (Promega, Madison, WI, USA) or MTT assay (Sigma-Aldrich, Milan, Italy), according to the manufacturer's instructions. 3×10^4^ senescent cells were seeded in triplicate into 24-well plates. 5×10^3^ proliferating cells were seeded in triplicate into 96-well plates. 16h later, cells were either treated with trabectedin or not treated. Cell viability was estimated 72h after drug washout.

### Escape from senescence

2×10^5^ senescent tumor cells were plated in triplicate in a 6-well plate. After 3 to 4 days, cells were treated or not with trabectedin (0.5 to 20 nM) for 24h, extensively washed, and incubated under standard conditions. Colonies that escaped senescence were stained with 1% Methylene Blue (Sigma-Aldrich, Milan, Italy) in 50%(v/v) ethanol, from 15 to 30 days after drug washout.

### mRNA quantification by real-time RT-PCR

Real-time RT-PCR was carried out with cDNAs reverse-transcribed from total RNA by using iScript cDNA Synthesis Kit (Bio-Rad, Segrate, MI, Italy) and amplified using iQ SYBR Green Supermix (Bio-Rad, Segrate, MI, Italy). Relative mRNA quantitation was performed by a comparative Ct method using Step-one software (Applied Biosystems, Australia).

The primers were:

IL-6: 5’-CCACTCACCTCTTCAGAACG-3’ and 5’-CATCTTTGGAAGGTTCAGGTTG-3’

IL-8: 5’-AAATTTGGGGTGGAAAGGTT-3’ and 5’-TCCTGATTTCTGCAGCTCTGT-3’;

TNF-α: 5’-ACTTTGGAGTGATCGGCC-3’ and 5’-GCTTGAGGGTTTGCTACAAC-3’;

CXCL10: 5’-ACTGGTTCAGCAGCCATCTT-3’ and 5’-TGCAGTCTACACAGCTTCGG-3’;IFN-γ:5’-GCATCGTTTTGGGTTCTCTTG-3’ and 5’-AGTTCCATTATCCGCTACATCTG-3’;

RANTES: 5’-TGTACTCCCGAACCCATTTC-3’ and 5’-TACACCAGTGGCAAGTGCTC-3’;

IL-1b: 5’-ATGCACCTGTACGATCACTG-3’ and 5’-ACAAAGGACATGGAGAACACC-3’;

IL-1a: 5’ –TGTATGTGACTGCCCAAGATG-3’ and 5’-TTAGTGCCGTGAGTTTCCC-3’;

Survivin: 5’-TAATACCAGCACTTTGGGAGG-3’ and 5’-GGCTCTTTCTCTGTCCAGTTTC-3’;

XIAP: 5’- GCACGGATCTTTACTTTTGGG-3’ and 5’- GGGTCTTCACTGGGCTTC-3’.

### Conditioned media preparation, proliferation assay and wound healing assay

Senescent tumor cells were treated with trabectedin, extensively washed and incubated for 3 to 4 days with normal culture medium. Subsequently, proliferating and senescent tumor cells were rinsed with serum-free medium and incubated in medium supplemented with 1% FBS for a further 48 hours. Conditioned media from proliferating and senescent tumor cells, either treated or not treated, were collected, filtered through sterile (0.2 μm) Millipore membranes and stored at -80°C. The volume of conditioned medium was normalized for cell number.

3×10^3^ proliferating tumor cells were seeded in triplicate into 96-well plates. 16h later cells were treated with conditioned media. Cell viability was determined after 72h using either CCK8 assay (Dojindo Molecular Technologies, Rockville, MD, USA), or MTS assay (Promega, Madison, WI, USA).

For wound healing assays, A549 cells were plated in duplicate in 12-well plates. When cells reached confluency, one linear scar was drawn in the monolayer by a white tip. Cells were washed and phase-contrast microscopy pictures were taken. The MRI Wound Healing Tool (ImageJ software) was used to measure the wound in two areas of the linear scar. The average of duplicate wells was calculated and used as time=0. Then, cells were treated with conditioned media or control media (1% FBS and 10% FBS). After 24h, the area of wounds was measured with MRI Wound Healing Tool. The average of duplicate wells was calculated, and wound closure was expressed as percent of time=0.

### Transwell migration assays

3×105 THP-1 cells were resuspended in 300μl of serum-free DMEM medium and were seeded in the upper compartment of the transwells (8 μm pore size, Corning Life Sciences, Tewksbury MA, USA), placed in 24-well plates. 500μl of either control media (DMEM supplemented or not with 10% FBS) or conditioned media were added to the lower compartment. After 3h incubation, cells that had migrated to the lower compartment through the filter were counted using a cytometer.

### Flow cytometry

Analyses of Fas/CD95 surface expression, cleaved Caspase-8, and measurement of mitochondrial membrane potential were performed as previously described [6]. The anti-Fas-PE (CD95; 555674) and anti-TRAIL-PE (CD-253; 550516) antibodies were purchased from BD Biosciences (Milan, Italy).

For cell cycle analyses, 6×105 proliferating and senescent cells were seeded into 60mm dishes. After 24h, cells were incubated with 10 μM 5-bromo-2-deoxyuridine (BrdU) for 30 min (proliferating) or 1h (senescent), fixed with ethanol and routinely kept at -20°C overnight. After two washes with phosphate-buffered saline (PBS), cells were incubated in 4M HCl for 30 min. Cells were washed with 0.1% Tween 20 in PBS, and incubated with FITC-conjugated anti-BrdU antibody (BD Biosciences, San Jose, CA, USA), according to the instructions of the manufacturer. Cells were washed with 0.1% Tween 20 in PBS, treated with 50μg/ml RNase DNase-free and 50μg/ml propidium iodide for 20 min, and analyzed using an Accuri C6 flow cytometer (BD Bioscience). Cell cycle distribution was analyzed by ModFit LT program (Verity Software House Inc., USA).

### Western blot analysis

Total cell proteins preparations and Western blots analyses were performed as previously reported [6]. The anti-p65 (C-20), IκB-alpha (C-15), p53 (DO-1), p21 (C-19), Bcl-XL (S18), Bcl-2 (100), C/EBP-beta (C-19) and Actin (I-19) antibodies were from Santa Cruz Biotechnology (Santa Cruz, CA, USA). The anti-cleaved Caspase-8 (Asp391), STAT3 (phospho-Tyr705), p44/42 MAPK (phospho-Thr202-Tyr204) and p44/42 MAPK antibodies were from Cell Signaling Technology (Danvers, MA, USA). The anti-α-Tubulin was from Serotec (Oxford, UK, MCAP77).

### Dual luciferase assay

Proliferating (8×10^4^) or senescent (4×10^4^) cells were seeded in 12-well plates. After 16h cells were cotransfected with 0.8 μg of Ig-κB-Luc reporter gene plasmid and 0.04 μg of pRL-SV40 plasmid (Renilla luciferase) using Lipofectamine 2000 (Thermo Fisher Scientific, MA, USA). Cells were treated with trabectedin and, after 24h, luciferase activity was measured using Dual-Luciferase Reporter Assay System (Promega, Madison, WI, USA,) and GloMax 20/20 Luminometer (Promega, Madison, WI, USA). Luciferase activity was expressed as relative activity (i.e. Firefly luciferase /Renilla luciferase).

Senescent MCF-7 cells were cotransfected with either Bcl-XL-κB-luciferase reporter plasmid and pRL-SV40 or Bcl-XL Δ-κB luciferase and pRL-SV40, in a 20:1 ratio, and treated as above. The Bcl-XL-κB-luc and Bcl-XL-Δ-κB-luc reporter plasmids were constructed by Dr. F. Aillet (University of Dandee, UK) and were kindly provided by Prof. R. Hay (University of Dandee, UK) and Prof. N. Perkins (Newcastle University, UK).

### Statistical analysis

Statistical significance was determined using an unpaired Student's t-test. p-values ≤0.05 were considered statistically significant. In all the manuscript: ^*^, p ≤ 0.05; ^**^, p ≤ 0.005; ^***^, p ≤ 0.0001.

## SUPPLEMENTARY MATERIALS FIGURES



## Abbreviations

TIS: Therapy-induced senescence
SASP: senescence-associated secretory phenotype
C.M.: conditioned media
CSC: cancer stem cell-like
EMT: epithelial-mesenchymal transition
FBS: fetal bovine serum
BrdU: 5-bromo-2-deoxyuridine
PBS: phosphate-buffered saline
