# Supplementary material for: Trabectedin modulates the senescence-associated secretory phenotype and promotes cell death in senescent tumor cells by targeting NF-κB
Source: Oncotarget. 2018 Apr 13;9(28):19929–44. doi: 10.18632/oncotarget.24961 (PMC5929437; doi:10.18632/oncotarget.24961)
Supplement: Supplementary file 1 [file oncotarget-09-19929-s001.pdf]

Trabectedin modulates the senescence-associated secretory phenotype and promotes cell death in senescent tumor cells by targeting NF-κB

SUPPLEMENTARY MATERIALS

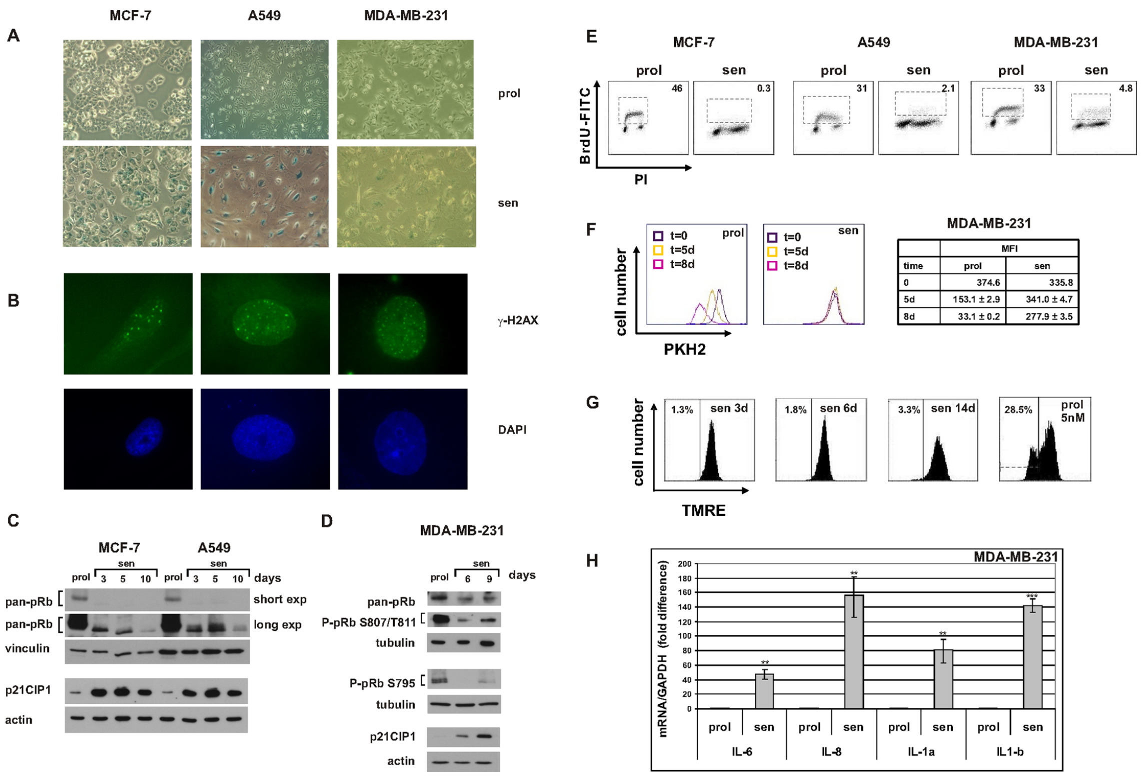

Supplementary Figure 1: Premature senescence in tumor cell lines. (A) MCF-7 and A549 cells were treated with doxorubicin (200 nM and 600 nM, respectively) for 72h. MDA-MB-231 cells were treated with 10μM cisplatin for 24h. Cells were extensively washed and morphological alterations and SA-beta-gal staining were analyzed 7 days after release from the drug. **(B)** MCF-7, A549 and MDA-MB-231 cells were treated as in (A). Seven days after induction of senescence cells were immunostained with an anti-gamma-H2AX monoclonal antibody followed by secondary fluorescein-conjugate antibodies. Nuclei were stained with DAPI. **(C)** Western blot analysis of pRb and $p21^{CIP1}$ in total cell lysates prepared from proliferating (prol) and doxorubicin-induced senescent (sen) MCF-7 and A549 cells, at indicated times after release from the drug. Filters were stripped and reprobed with anti-vinculin and anti-actin antibodies, as loading control. **(D)** Western blot analysis of pRb, phospho-Ser807/Thr811-Rb, phospho-Ser795-Rb and $p21^{CIP1}$ in total cell lysates prepared from proliferating (prol) and cisplatin-induced senescent (sen) MDA-MB-231 cells, at indicated times after release from the drug. Filters were stripped and reprobed with anti-tubulin and anti-actin antibodies, as loading control. **(E)** Representative flow cytometric data. Premature senescence was induced as in (A). Five (MCF-7 and A549) and seven (MDA-MB-231) days after induction of senescence, proliferating and senescent cells were incubated with 5-bromo-2-deoxyuridine (BrdU). The number of BrdU-labelled cells was determined and the percentage is shown in the figure. **(F)** *Left*: representative flow cytometric data. MDA-MB-231 cells were treated with 10μM cisplatin for 24h, extensively washed, labeled with the lipophilic dye PKH2, and analyzed at indicated times after release from the drug. Proliferating cells were labeled at the same time. PKH2 fluorescence intensity was compared with the intensity of the starting population (t=0, blue line). *Right*: PKH2 mean fluorescence intensity (MFI) for proliferating (prol) and senescent (sen) MDA-MB-231 cells analyzed at indicated times. Data are mean±S.D. of three independent samples. **(G)** Representative flow cytometric data. MDA-MB-231 cells were treated with 10μM cisplatin for 24h. Mitochondrial membrane depolarization was estimated by TMRE staining at indicated times after release from the drug in senescent (sen) cells. Proliferating (prol) MDA-MB-231 cells were induced to undergo apoptosis by treatment with 5 nM trabectedin for 2h and were analyzed after 72h, as positive control. **(H)** SASP induction in drug-induced senescent MDA-MB-231. MDA-MB-231 cells were treated with 10μM cisplatin for 24h. Cytokines expression was analyzed in proliferating (prol) and senescent (sen) cells by real-time PCR analysis 7 days after release from the drug. Data are mean±S.D. of three independent experiments.

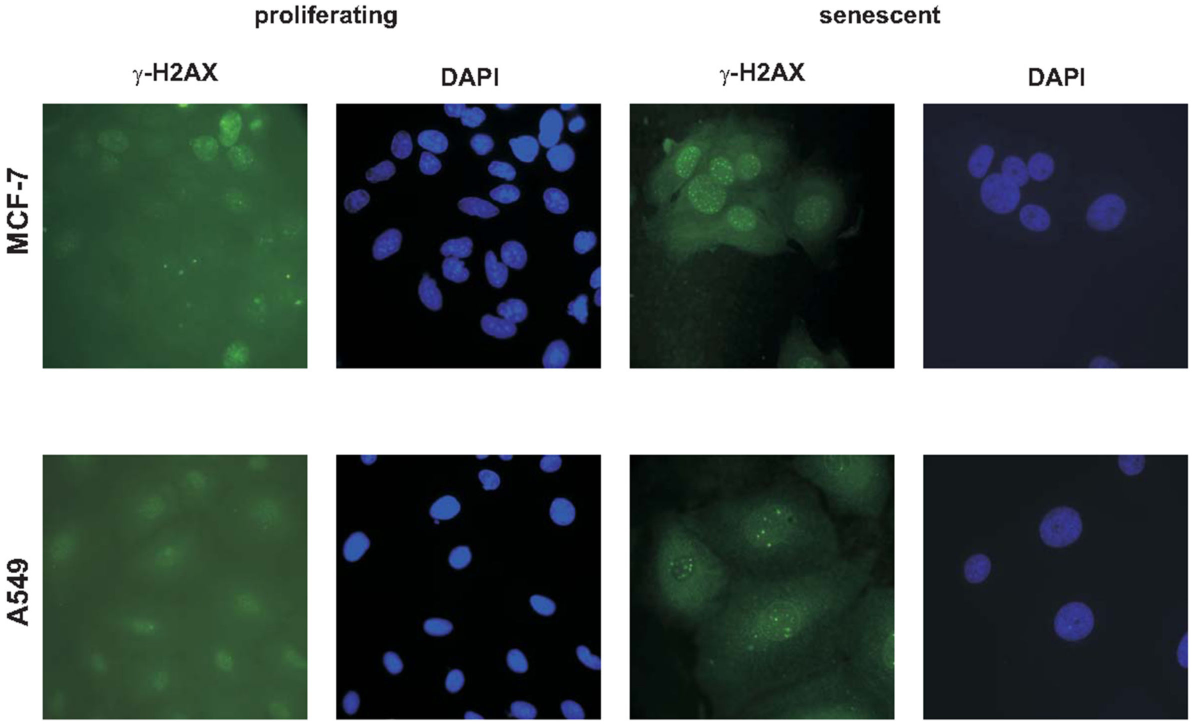

Supplementary Figure 2: gamma-H2AX staining in proliferating and senescent tumor cells. Proliferating MCF-7 and A549 cells were immunostained with an anti-gamma-H2AX monoclonal antibody followed by secondary fluorescein-conjugate antibodies. MCF-7 and A549 cells were treated with doxorubicin (200 nM and 600 nM, respectively) for 72h to induce senescence. Height days after induction of senescence cells were immunostained with an anti-gamma-H2AX monoclonal antibody followed by secondary fluorescein-conjugate antibodies. Nuclei were stained with DAPI.

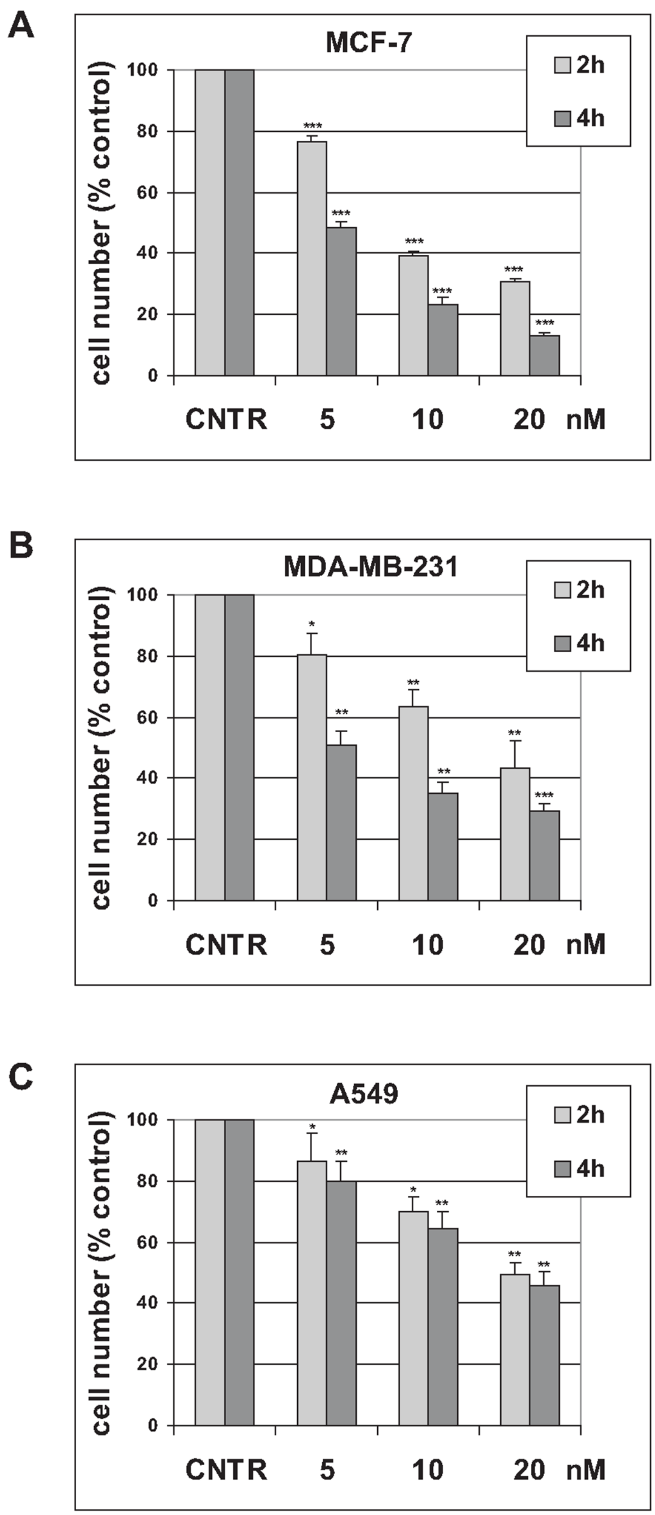

Supplementary Figure 3: Effect of trabectedin on tumor cells viability. MCF-7 and A549 cells were induced to undergo senescence by treatment with doxorubicin. MDA-MB-231 cells were induced to undergo senescence by treatment with cisplatin. Four days after release, senescent cells were treated with 5 to 20 nM trabectedin for 72h. Cells were counted 72h after trabectedin washout. Data are mean ± S.D. of one experiment (A549) or two independent experiments (MCF-7, MDA-MB-231), performed in triplicate.

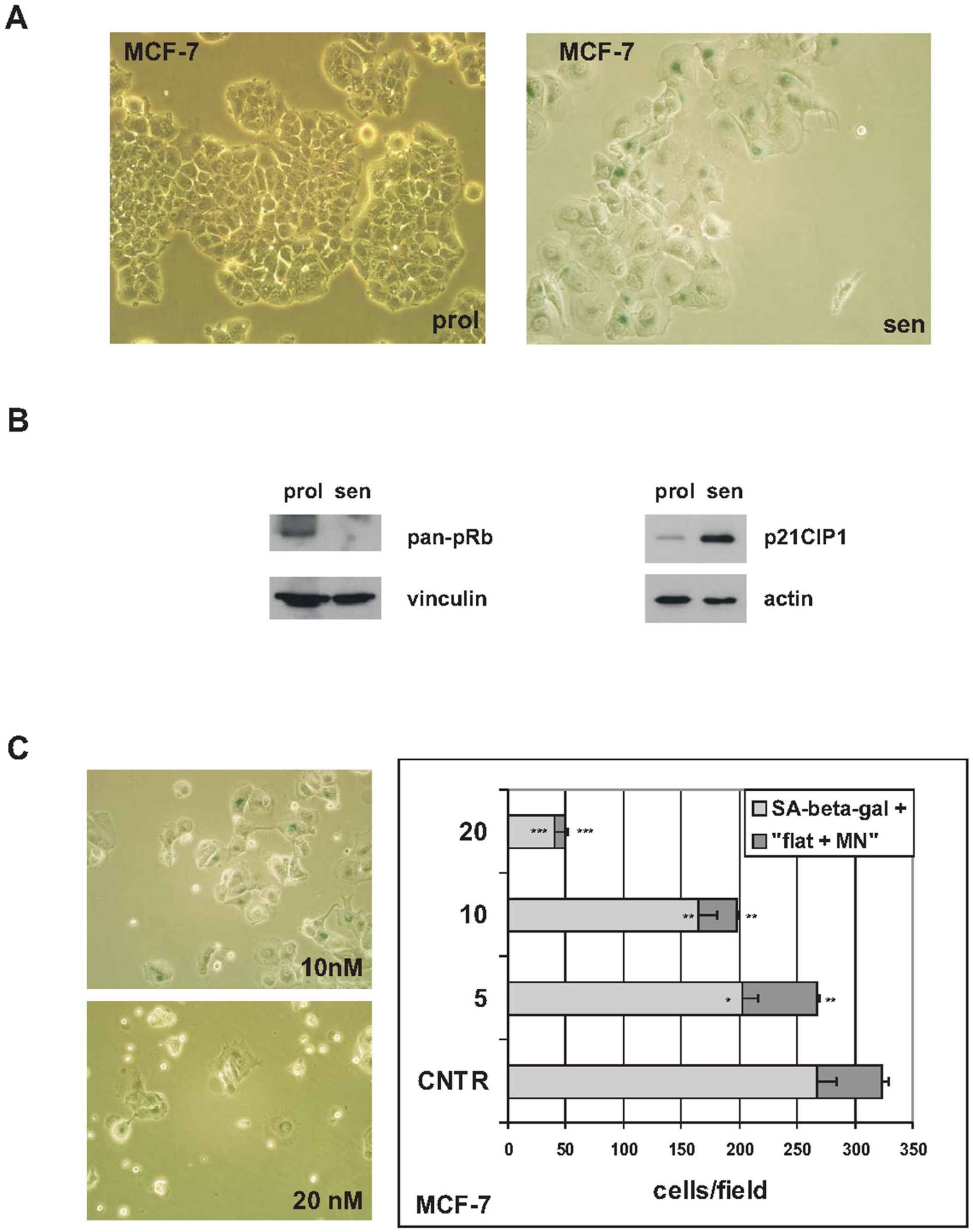

Supplementary Figure 4: Hydrogen peroxide-induced senescence in MCF-7 cells. (A) MCF-7 cells were treated with 500µM H2O2 for 2h. Cells were extensively washed and morphological alterations and SA-beta-gal staining were analyzed 6 days after release. **(B)** Western blot analysis of pRb and p21[CIP1] in total cell lysates prepared from proliferating (prol) and H2O2-induced senescent (sen) MCF-7 cells, six days after senescence induction. Filters were stripped and reprobed with anti-vinculin and anti-actin antibodies, as loading control. **(C)** Six days after H2O2 exposure, senescent MCF-7 cells were treated with 5 to 20 nM trabectedin for 2h and analyzed 72h after drug washout. *Left*: cell death in senescent MCF-7 cells was examined by morphologic changes under a phase-contrast microscope, after SA-beta-gal staining. *Right*: Cell morphology and SA-gal activity were analyzed by phase-contrast microscopy. The percentage of cells with altered cellular morphology, such as flattening and micronucleation (flat + MN), or positive SA-beta-gal staining (SA-beta-gal +), was determined by counting six random fields for each sample. For each determination a minimum of 200 cells were counted.

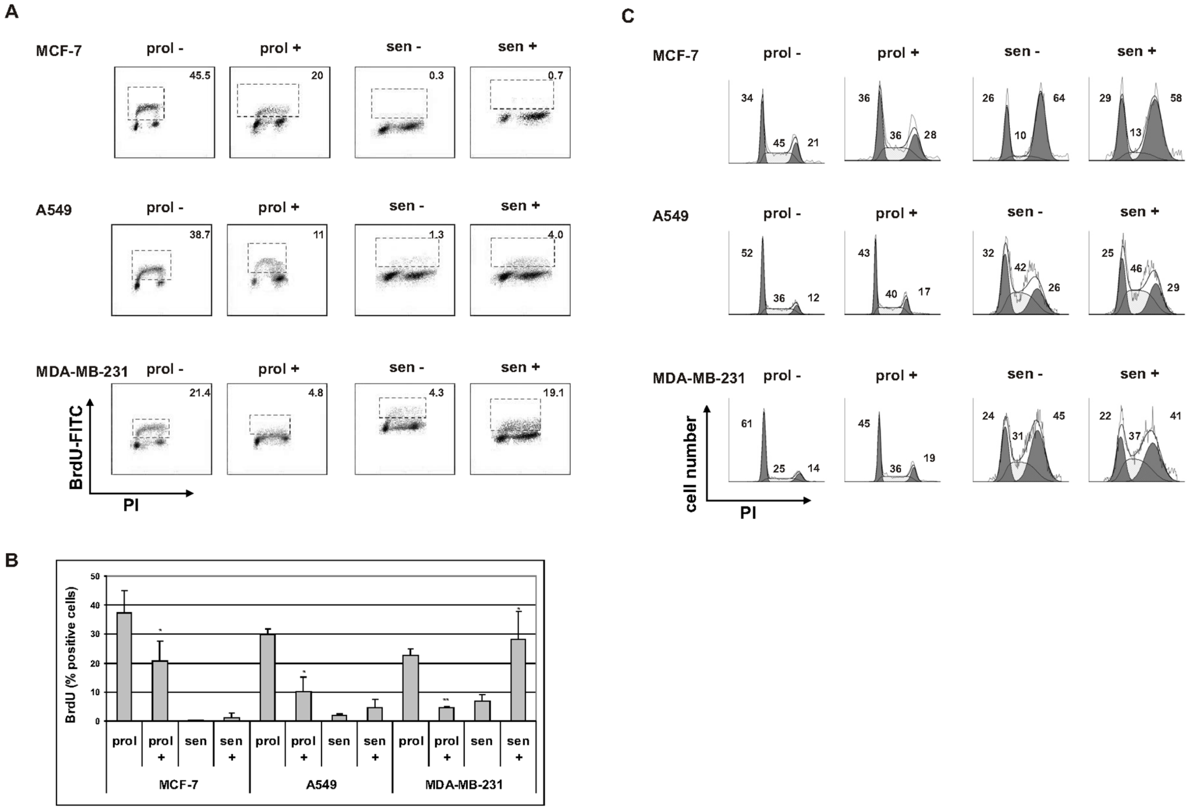

Supplementary Figure 5: Cell cycle distribution in trabectedin-treated senescent tumor cells. (A) Representative flow cytometric data. Four days after induction of senescence, MCF-7 and MDA-MB-231 cells were treated with 10 nM trabectedin for 2h. Six days after induction of senescence, A549 cells were treated with 10 nM trabectedin for 4h. Proliferating and senescent cells were incubated with 5-bromo-2-deoxyuridine. The number of BrdU-labelled cells was determined and the percentage is shown in the figure. **(B)** BrdU incorporation in proliferating (prol) and senescent (sen) tumor cells, either untreated or treated with trabectedin (+). Bar graph summarizes the data (mean ± S.D.) from three independent experiments. **(C)** Representative flow cytometric data. Cell cycle distribution in proliferating (prol) and senescent (sen) tumor cells, either untreated (-) or treated with trabectedin (+) was analyzed by flow cytometry following propidium iodide staining. The percentage of cells in G1, S, or G2/M phase is given.

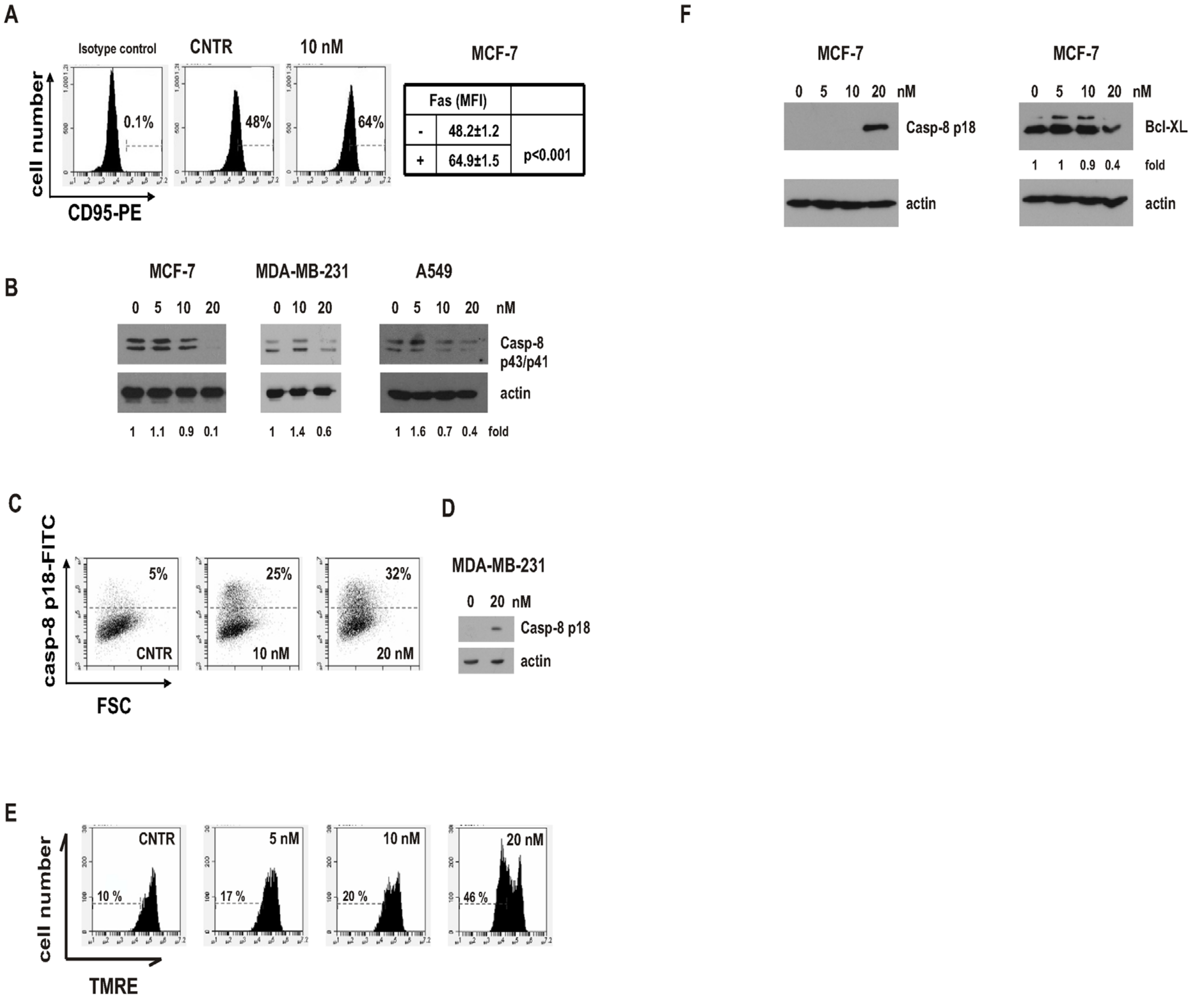

Supplementary Figure 6: Trabectedin induces apoptosis in senescent tumor cells. **(A)** Representative flow cytometric data. Four days after induction of senescence, MCF-7 cells were treated with 10 nM trabectedin for 1h and analyzed for Fas expression 72h after drug washout. The levels of surface Fas in untreated senescent cells (-) and trabectedin treated senescent cells (+) are expressed in table as the mean fluorescence intensity (MFI) minus background fluorescence of isotype-matched control. Data are mean±S.D. of three independent experiments. Statistical analysis by unpaired t-test: $p \leq 0.001$. **(B)** Four days after induction of senescence, cells were treated with indicated concentrations of trabectedin (MCF-7 1h, MDA-MB-231 2h, A549 4h treatment). Processing of p43/p41 Caspase-8 fragments was detected by Western blot analysis on total cell lysates, 72h after drug washout. Filters were stripped and re-probed with anti-actin antibody. p43/p41 Casp-8 levels, normalized to the relative actin levels, are reported as fold change of relative untreated sample. **(C)** Representative flow cytometric data. Four days after induction of senescence, MCF-7 cells were treated with indicated concentrations of trabectedin for 4h and Casp-8 p18 was detected 24h after drug washout. **(D)** MDA-MB-231 cells were treated with 10μM cisplatin for 24h. Five days after induction of senescence, MDA-MB-231 cells were treated with 20 nM trabectedin for 4h. Formation of Casp-8 p18 was detected by Western blot analysis on total cell lysates, 48h after drug washout. Filters were stripped and re-probed with anti-actin antibody. **(E)** Representative flow cytometric data. Four days after induction of senescence, MCF-7 cells were treated with indicated concentrations of trabectedin for 1h. Mitochondrial membrane depolarization was estimated by TMRE staining 72h after drug washout. **(F)** Three days after release from H2O2, senescent MCF-7 cells were treated with trabectedin for 2h. Casp-8 and Bcl-XL proteins were detected 72h after drug washout. Filters were stripped and re-probed with anti-actin antibody, as loading control. Bcl-XL levels, normalized to the relative actin levels, are reported as fold change of untreated cells.

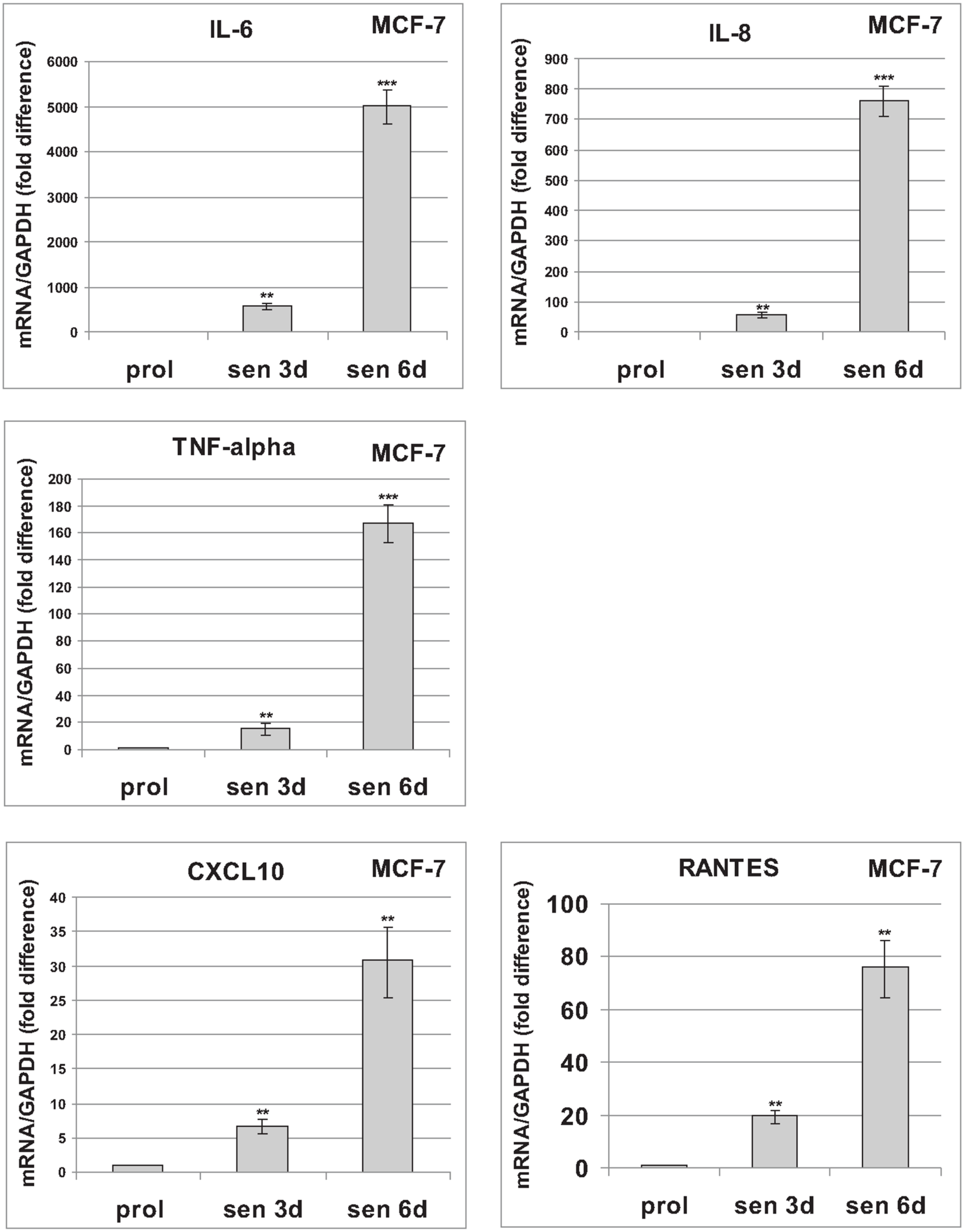

Supplementary Figure 7: SASP develops gradually in drug-induced senescent MCF-7 cells. Premature senescence was induced by treating MCF-7 cells with doxorubicin (200 nM) for 72h. Expression of cytokine/chemokine was measured by real-time PCR in proliferating (prol) and senescent (sen) cells 3 days and 6 days after release from the drug. Data are mean ± S.D. of one representative experiment out of two independent experiments performed in triplicate.

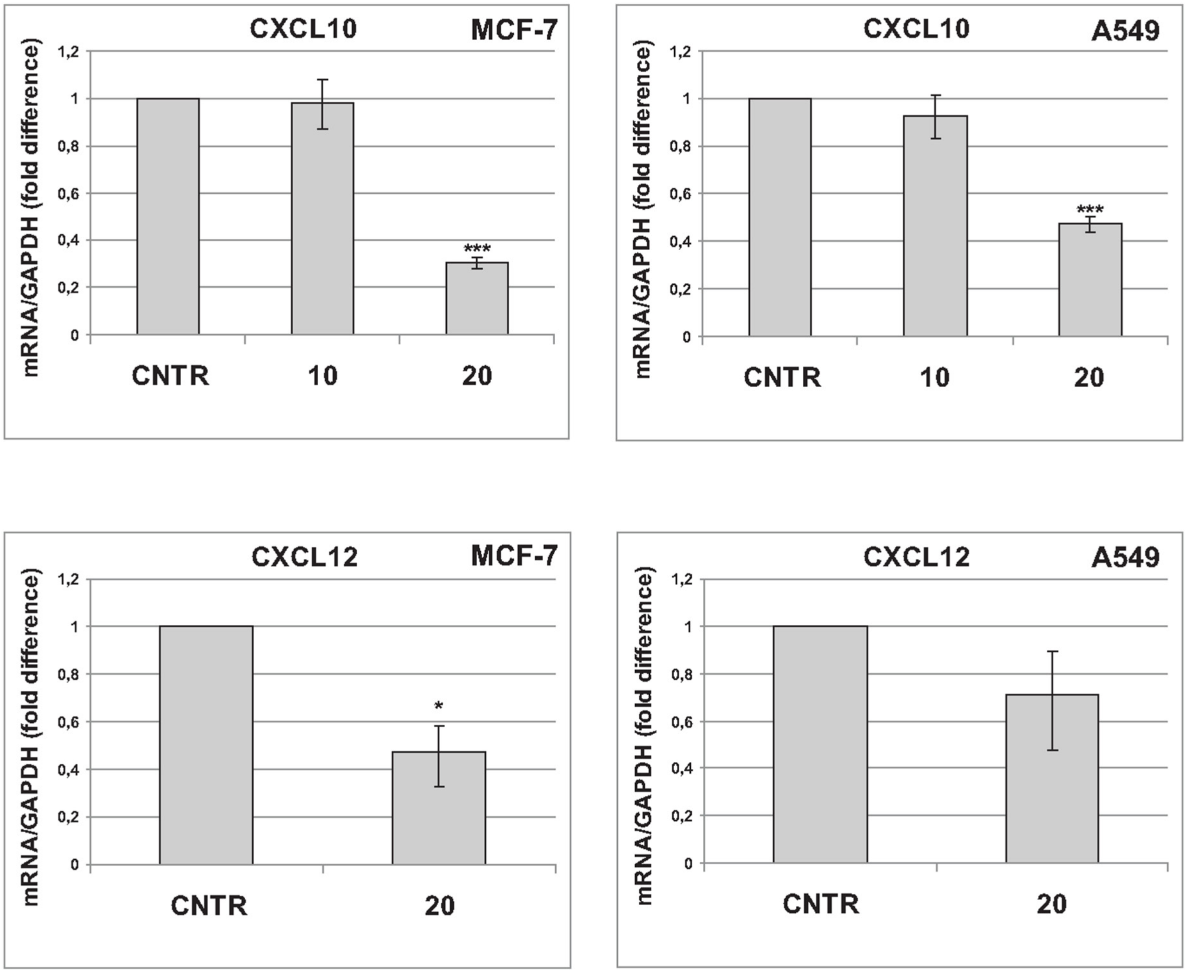

Supplementary Figure 8: Four days after induction of senescence, MCF-7 and A549 cells were treated with indicated nanomolar concentrations of trabectedin for 1h or 2h, respectively. CXCL10 and CXCL12 expression was analyzed by real-time PCR 72h after drug washout. Data are mean±S.D. of three independent experiments.

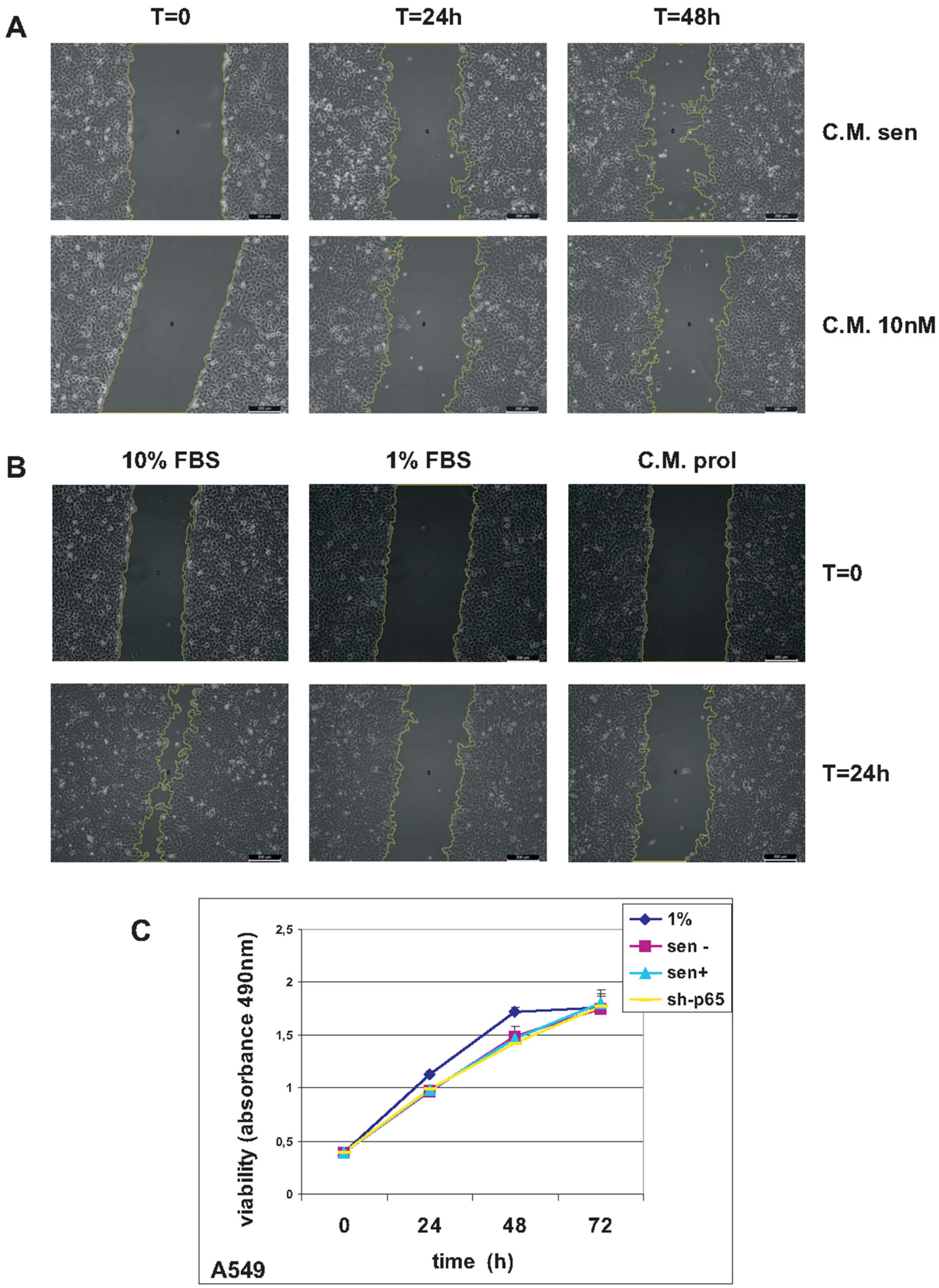

Supplementary Figure 9: (A) Representative pictures from wound healing assay. Proliferating A549 cells were incubated with C.M. from untreated (C.M. sen) and trabectedin-treated (C.M. 10 nM) senescent cells for 24 and 48h. **(B)** Representative pictures from wound healing assay. Proliferating A549 cells were incubated with DMEM 10% FBS, DMEM 1% FBS or C.M. from proliferating A549 cells (C.M. prol) for 24h. **(C)** Effects of conditioned media from untreated senescent (sen-), trabectedin-treated senescent (sen+) or RelA/p65-interfered senescent A549 cells (sh-p65) on cell viability. Proliferating A549 cells were incubated with C.M. and cell viability was determined by MTS assay. DMEM supplemented with 1% FBS was used as control. Data are mean±S.D. of three independent experiments.

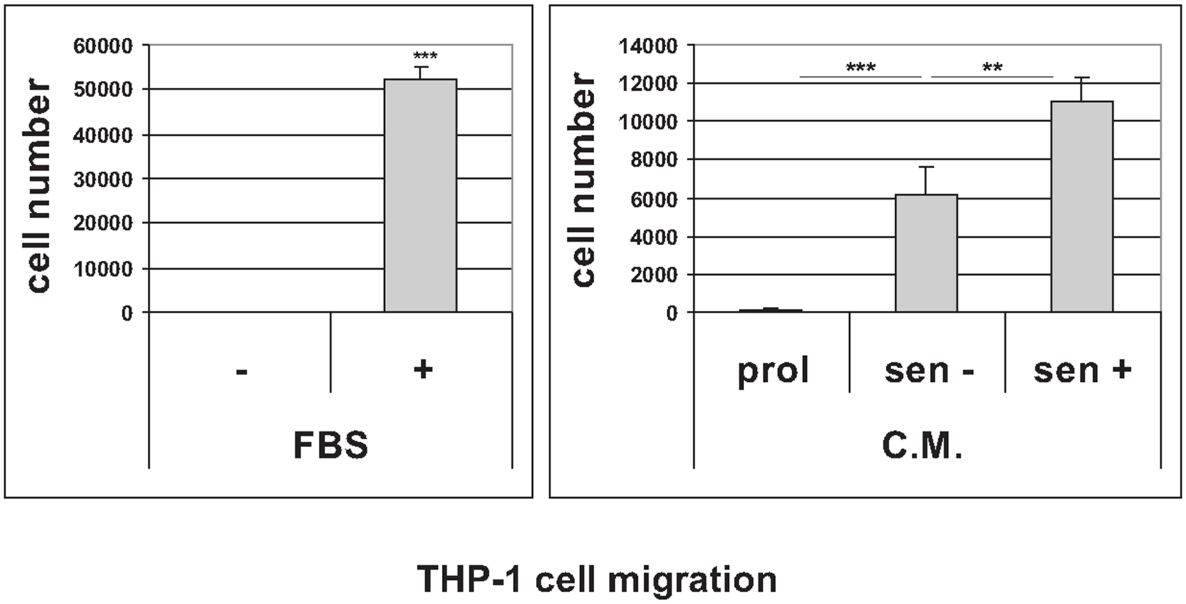

Supplementary Figure 10: Trabectedin modulates conditioned media-induced monocyte migration. THP-1 monocyte migration stimulated by conditioned media (C.M.) from proliferating MCF-7 cells (prol), untreated senescent cells (sen-) or 10 nM treated senescent cells (sen+). Data are mean ± S.D. of one representative experiment out of three independent experiments performed in triplicate. DMEM supplemented (+) or not (-) with 10% FBS was used as positive or negative control, respectively.

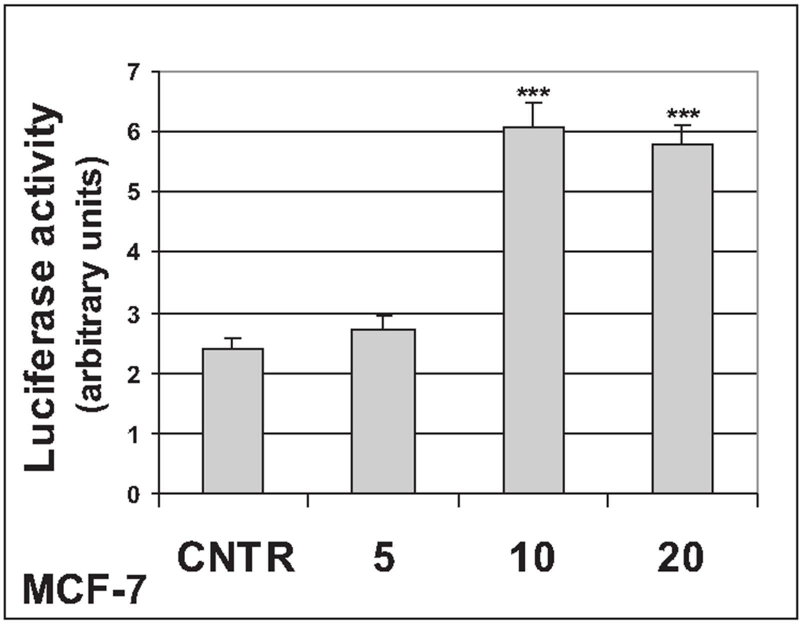

Supplementary Figure 11: Proliferating MCF-7 were transiently cotransfected with Ig-κB luciferase reporter and Renilla luciferase reporter plasmids. After 24h, cells were either untreated (CNTR) or treated with trabectedin (5 to 20 nM) for 1h. Luciferase activity was measured 24h after drug washout. Data are mean ± S.D. of one representative experiment performed in triplicate.

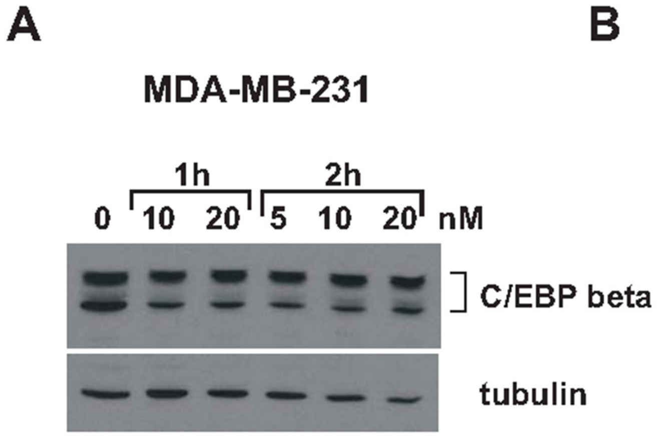

Supplementary Figure 12: Effect of trabectedin on C/EBP-beta protein expression. **(A)** Proliferating MDA-MB-231 cells were treated for 1h or 2h with indicated concentrations of trabectedin. C/EBP-beta was detected by Western blot analysis on total cell lysates, 72h after drug washout. Filters were stripped and reprobed with anti-α-tubulin antibodies as a loading control. **(B)** MDA-MB-231 cells were treated with 10μM cisplatin for 24h. Five days after induction of senescence, MDA-MB-231 cells were treated with 20 nM trabectedin for 4h. C/EBP-beta was detected in untreated proliferating cells and in senescent cells, 48h after drug washout. Filters were stripped and re-probed with anti-actin antibody.
